# Atmospheric Pressure Plasma Deposition of TiO_2_: A Review

**DOI:** 10.3390/ma13132931

**Published:** 2020-06-30

**Authors:** Soumya Banerjee, Ek Adhikari, Pitambar Sapkota, Amal Sebastian, Sylwia Ptasinska

**Affiliations:** 1 Radiation Laboratory, University of Notre Dame, Notre Dame, IN 46556, USA; sbanerjee424@gmail.com (S.B.); eadhikar@alumni.nd.edu (E.A.); Pitambar.Sapkota.2@nd.edu (P.S.); asebast1@nd.edu (A.S.); 2 Department of Physics, University of Notre Dame, Notre Dame, IN 46556, USA

**Keywords:** atmospheric pressure plasma, titanium dioxide, thin film deposition

## Abstract

Atmospheric pressure plasma (APP) deposition techniques are useful today because of their simplicity and their time and cost savings, particularly for growth of oxide films. Among the oxide materials, titanium dioxide (TiO_2_) has a wide range of applications in electronics, solar cells, and photocatalysis, which has made it an extremely popular research topic for decades. Here, we provide an overview of non-thermal APP deposition techniques for TiO_2_ thin film, some historical background, and some very recent findings and developments. First, we define non-thermal plasma, and then we describe the advantages of APP deposition. In addition, we explain the importance of TiO_2_ and then describe briefly the three deposition techniques used to date. We also compare the structural, electronic, and optical properties of TiO_2_ films deposited by different APP methods. Lastly, we examine the status of current research related to the effects of such deposition parameters as plasma power, feed gas, bias voltage, gas flow rate, and substrate temperature on the deposition rate, crystal phase, and other film properties. The examples given cover the most common APP deposition techniques for TiO_2_ growth to understand their advantages for specific applications. In addition, we discuss the important challenges that APP deposition is facing in this rapidly growing field.

## 1. Introduction

Plasma is defined as fully or partially ionized gas with many unique properties attributable to the long-range electromagnetic interaction between charged species. It consists of electrons, excited heavy species such as ions and neutrals, electromagnetic radiation (i.e., photons), and electromagnetic fields. Further, plasma possesses a quasi-neutral property, one of the unique properties of plasma as a state of the matter, which means that it is electrically neutral with approximately equal numbers of positively and negatively charged species. Plasma is classified into two groups based on temperature and equilibrium conditions: (1) thermal or equilibrium plasma, and (2) non-thermal or non-equilibrium plasma [1]. A thermal plasma is characterized by a high level of ionization, and all charged species achieve thermal equilibrium at a high temperature (~10^4^ K), while a non-thermal plasma, also referred to as a cold plasma, is characterized by weak ionization, and the electrons are not in thermal equilibrium with heavy species. Therefore, the gas temperature in cold plasma is equivalent to room temperature or slightly above, and ranges from approximately ~40 °C to ~150 °C. The electron density of cold plasma is typically less than 10^19^ m^−3^ [2]. Non-thermal plasmas can be generated at both low pressure and atmospheric pressure. Overall, the temperature of each plasma species is usually defined based on the average kinetic energy of the individual species:

(1) $$T_{i}=\frac{2}{3K_{B}}\langle E_{i}\rangle ,\;i=1,\;2,\;3,\;\ldots$$

in which *K_B_* is the Boltzmann constant, *E_i_* is the kinetic energy of an individual species (*i*) in thermal motion, and the angular brackets denote averaging over the entire number of species. In plasma physics, such temperatures are commonly measured in electron volts: 1 eV ≃ 11600 K [3]. For more detailed information on weakly ionized plasmas, we refer the reader to the most recent review article that covers their basic principles and applications [4].

Thermal (equilibrium) plasmas have been used for material processing for more than 100 years. One common example of thermal plasma in material processing is electric welding. In general, most of the electrical power used to ignite plasma is converted into heat, and, therefore, thermal plasmas can melt metallic surfaces as well as the electrodes used to ignite the plasma. However, it is undesirable to have such excessive heat in contact with the heat-sensitive or softer surfaces used in material processing, such as coating, cleaning, and etching. For these applications, cold plasmas may be a good alternative, as the electrons have high thermal energy, while the heavier species remain “cold”. Thus far, large-scale industrial technology is generally based on non-thermal plasmas ignited at reduced or low gas pressures, in which the neutral gas temperature is low enough to prevent damage to processing materials. There is also a long history of using non-thermal (non-equilibrium) plasmas in material processing; however, using atmospheric pressure plasmas (APPs) to do so is recent. This novel approach to material processing is appealing because of the combination of simplicity, low cost, and the wide range of possibilities for surface treatment and modification [5,6]. Therefore, there has been a rapidly growing interest in recent years in replacing a low-pressure plasma with a cold atmospheric plasma, as expensive vacuum equipment can be eliminated. Currently, there are several methods to generate APPs from microwave or radio frequency (rf) discharge, dielectric barrier discharge (DBD), and direct current glow discharge [5,7]. These methods use various gases or gas mixtures as a feed gas and have unique features that are suitable for numerous applications. For example, a process of plasma-enhanced/assisted chemical vapor deposition (PECVD/PACVD; here we will use the PECVD term hereafter) at atmospheric pressure can be optimized by using a mixture of various gases as well as a gas/liquid precursor in the feed gas to enhance plasma chemistry [8]. The scientific and industrial communities recognize the advantage of using APP already because of rich plasma chemistry, which plays a significant role in material processing techniques, such as PECVD, plasma-assisted etching, and plasma polymerization [1,9,10,11,12]. Plasma can effectively deliver reactive chemical species that promote surface reactions that would otherwise require a high substrate temperature. Thus, APPs are efficient sources to achieve a high deposition rate of thin film at a low substrate temperature, which prevents any thermal damage or other side effects related to heating [6]. Lower substrate temperatures in PECVD are important in electronic applications in which a film is deposited onto device structures with low thermal budgets, particularly in those in which substrates are sensitive to high surface temperatures, such as polymers or organic compounds. Defect-free and strongly adherent thin film deposited over microscopic features is essential in manufacturing microelectronic devices because device structures are sensitive to temperature. Thus, due to the non-equilibrium nature of non-thermal plasmas, films can be deposited at low temperatures with a chemical composition and crystalline morphology that would be unattainable under higher-temperature equilibrium conditions [13].

## 2. Advantages of Atmospheric Pressure Plasma-Enhanced Deposition 

APP discharge is not only a relatively novel and versatile technique but is also an emerging tool in cost-effective solutions for many processing challenges in material sciences. Compared to compact deposition methods under vacuum conditions, APP-based deposition is a more robust technology that allows thin films to be deposited on substrates with various sizes regardless of their geometry [7]. Plasma-enhanced deposition techniques offer the benefit of minimal chemical waste throughout the process and are solvent-free compared to other methods, such as the sol-gel process. Moreover, APP deposition features relatively high deposition rates; however, its efficacy in achieving high deposition rates of coating depends upon numerous parameters, including optimal gas flow rates, concentrations of reactive species, plasma power, and precursors, as well as the type of deposition method used [7]. One of the best APP deposition methods that uses precursors and offers a significant rate of deposition is injection plasma processing [14]. Precursors, in the form of powder or volatile molecules, are introduced downstream of the plasma discharge, where they are vaporized and then interact with reactive species in the plasma discharge. The interaction of precursor molecules with highly reactive species in plasma lowers the activation energy required for deposition. As a result, deposition is achieved when the resulting mixture is sprayed onto the substrate, and the rate of deposition achieved by this method is usually on the order of 10 µm/min. 

In the case of titanium dioxide or titania (TiO_2_) deposition by an injection method, several works [15,16,17,18] have reported the use of different TiO_2_ solution precursors, but, in numerous studies, Ti-based compounds were used as precursors, among which the most widely used compound is titanium tetraisopropoxide (Ti[OCH(CH_3_)_2_]_4_, TTIP). Other precursors used commonly are titanium tetrachloride (TiCl_4_), titanium ethoxide (Ti_4_(OCH_2_CH_3_)_16_, Ti(OEt)_4_, TEOT), and titanium bis(acetylacetonate) diisopropoxide ([(CH_3_)_2_CHO]_2_Ti(C_5_H_7_O_2_)_2_, TIPO). However, contrary to other precursors, the former produces toxic by-products and chlorine contamination, and, therefore, is now used less frequently, while the two latter precursors, TEOT and TIPO, possess some advantages, such as low carbon content and relatively high resistance to water, respectively. It has been found that deposition rates using these precursors can even range from 20 to 40 µm/s [16], which is significantly higher than those obtained in traditional deposition techniques. Typically, deposition rates for conventional deposition techniques range on the order of nm/min [19]. For example, the deposition rate of TiO_2_ films using PECVD is in the range of 1 µm/min, which is roughly three orders of magnitude greater than conventional deposition methods [20]. 

Moreover, to achieve effective and efficient photocatalytic TiO_2_ coating, it is imperative to prevent the anatase phase from transforming to rutile. Research has shown that the anatase-to-rutile phase ratio may be tuned by varying the parameters, e.g., the arc current in plasma [21]. Studies have also reported that the transition from the anatase to rutile phase can be controlled and delayed at high temperatures for TiO_2_ deposited employing injection plasma processing [16].

**Figure 1:**
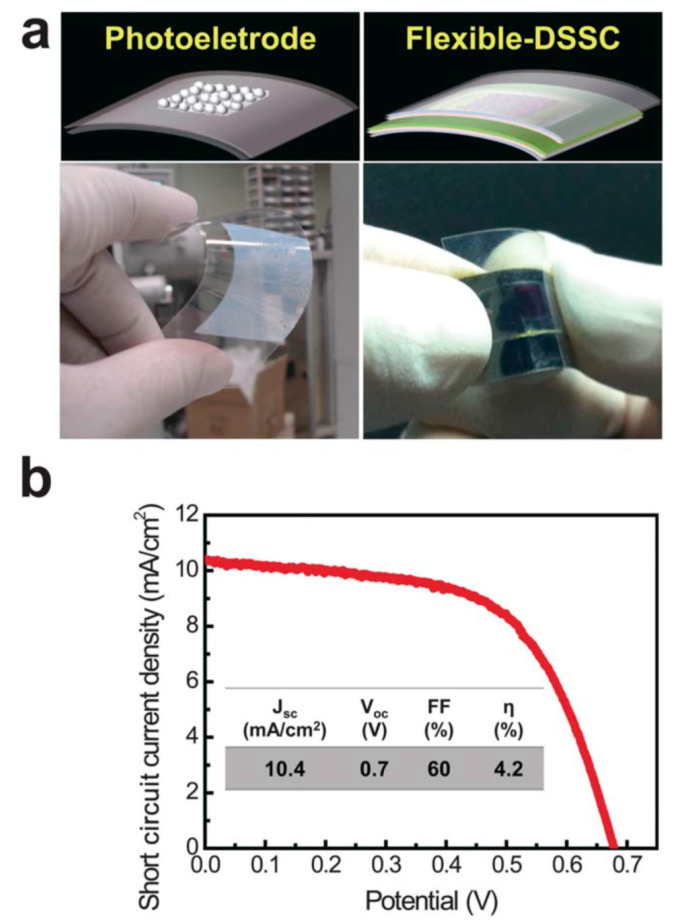
Flexible photoelectrode and DSSC fabricated by the APP process. (**a**) Schematic illustrations and real photo images of the flexible TiO_2_ photoelectrode and DSSC based on a tin-doped indium oxide (In_2_O_3_:Sn)-coated polyethylene naphthalate substrate. (**b**) Current density–voltage characteristics of the flexible DSSC under 1 sun illumination. The photovoltaic properties (J_SC_—the short-circuit current density, V_OC_—the open circuit voltage, FF—the fill factor, and η—energy conversion efficiency) of the flexible DSSC with a TiO_2_ film thickness of 3 μm are summarized in the inset of (**b**). Republished with the permission of the Royal Society of Chemistry, from [22]; permission conveyed through Copyright Clearance Center, Inc.

Another study showed that APP-based deposition of anatase TiO_2_ nanoparticles can be fabricated on a flexible, transparent plastic substrate to produce dye-sensitized solar cells (DSSCs) at low temperature (190 °C) [22]. Moreover, it has been demonstrated that this methodology can be scaled up to treat large areas (up to several meters), and, thus, it can be considered compatible with high-throughput, roll-to-roll manufacturing [22]; see Figure 1.

Specific descriptions of APP benefits are presented below for the specific methodologies, including PECVD, plasma-enhanced atomic layer deposition (PEALD), and atmospheric pressure dielectric barrier discharge deposition (APDBDD).

## 3. Importance of TiO_2_

Thin-film capacitors, antireflection coatings, optical waveguides, photo-electrochemical (PEC) cells, sensors, and mechanical or corrosion-resistant barriers are some selected applications of TiO_2_. Further, because of miniaturization in data-storage technology, the thickness of the oxide layer of dynamic random-access memory (DRAM) needs to be reduced, which will then, in turn, limit the use of silicon dioxide or silica (SiO_2_). Research has demonstrated that there is an abrupt increase in leakage current if SiO_2_ is thinner than 1.5 nm [23]. This is attributable to the low dielectric constants of SiO_2_ (~3.8), and, therefore, materials with a higher dielectric constant are required to replace silica. Highly dielectric materials, such as tantalum oxide (Ta_2_O_3_), aluminum oxide (Al_2_O_3_), yttrium oxide (Y_2_O_3_), barium/strontium titanate (Ba/Sr)TiO_3_, lead zirconate titanate (Pb(Zr,Ti)O_3_), and TiO_2_ have been studied for the past decade in search of a material with a low leakage current and closed packing density. Among these oxides, TiO_2_ stands out because of its high dielectric constant (i.e., 30–100) as well as its good thermal stability and sufficient barrier height.

In addition to the generic high dielectric property of TiO_2_, it can be used in photocatalysis, and has a high hydrophilicity and water splitting capability. These properties are related to different amounts of crystalline phases present in the deposited layer [24]. Therefore, depending on the material structure, TiO_2_ can be exploited for different applications related to energy conversion and catalysis.

Generally, TiO_2_ can be found in three crystalline allotropes: anatase, rutile, and brookite. The formation of these different crystal phases depends on the preparation methods, temperature, deposition parameters, types of substrate, and doping [24,25]. For example, TiO_2_ in the anatase crystalline phase, which is an excellent photocatalytic material, converts to rutile if annealed at higher temperatures. It is generally accepted that the pure anatase phase displays a higher photocatalytic activity compared to rutile TiO_2_ as was also supported theoretically by the exciton transport model [26]. High activity and the non-toxic properties of anatase TiO_2_, together with its exceptionally high chemical stability, make this compound ideal as a protective layer in PEC cells [27].

Lastly, anatase TiO_2_ can be a suitable material for photo-assisted water splitting in a solar cell upon exposure to light with energy greater than the TiO_2_ band gap (i.e., 3.26 eV) [28]. It is generally accepted that the formation of charge carriers by photon absorption initiates the redox reactions at the surface [29]. Photons with sufficient energy can create an electron–hole pair, and, as a result, the excited electron in the conduction band reduces oxygen to superoxide, while the hole in the valence band oxidizes water molecules to hydroxyl ions [30]. Moreover, titania has high durability as well as chemical activity and stability under ultraviolet (UV) light [31,32], which, in addition to its water splitting ability, makes it useful for antimicrobial treatment, water purification, self-cleaning purposes [33,34,35,36], and material passivation [37]. The species formed through redox reactions at the surface are sufficiently powerful to decompose organic molecules or microbes, leading to the self-cleaning ability of TiO_2_. Consequently, there is the disadvantage of a wide band gap in TiO_2_, as it cannot be used in solar cells for visible light. To overcome this issue, the study of titanium oxide band gap engineering by doping with metallic or non-metallic atoms has been initiated [38]. For example, research has found that nitrogen doping can improve visible-light photoactivity by reducing the band gap [39]. Thus, in recent years, more studies have focused on using APPs to deposit nitrogen-doped thin films: titanium oxynitride (TiO_x_N_y_) [40,41] and N–doped TiO_x_ [42,43]. 

Many important studies have been conducted on the properties of TiO_2_ thin films and nanostructures that make this material versatile for many different applications. We refer the reader to review articles published within the last year that have summarized work on TiO_2_ characterization and applications in energy- and environment-related processes [44,45,46,47].

## 4. Atmospheric Pressure Plasma-Enhanced Deposition Methods

**Figure 2:**
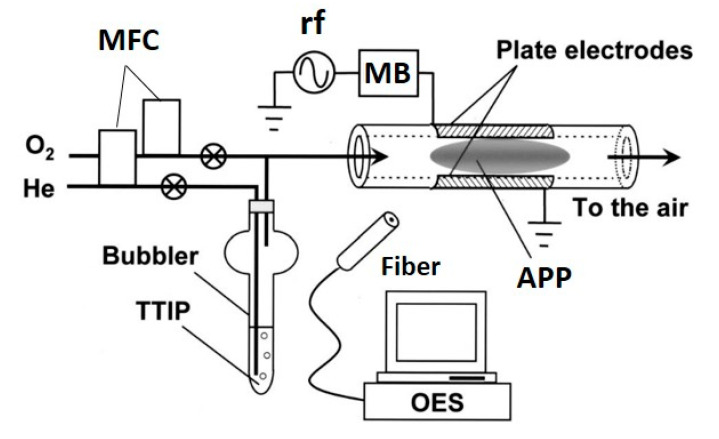
Schematic of the experimental set-up for TiO_2_ deposition on the inner surface of a quartz tube using PECVD. Parallel-plate electrodes were attached to the outer surface of the quartz tube. One electrode was connected to an rf (13.56 MHz) generator via a matching box (MB), while the other was grounded. One end of the quartz tube was connected to a gas line that was heated to 40 °C to prevent condensation of TTIP vapor during transfer. The gas flow to the tube was adjusted by mass flow controllers (MFCs). The other end of the quartz tube was open to the air; thus, in this experiment, APP, specifically microplasma, was generated. Light emitted from the APP was collected by an optical fiber and monitored by optical emission spectroscopy (OES). Reprinted with permission from Ref. [49]. Copyright 2008, American Vacuum Society.

This review focuses mainly on the use of APP-enhanced deposition methods for TiO_2_ films. However, some pioneering works are also reported and are followed by current research findings using plasma deposition methods, such as PECVD, PEALD and APDBDD, including deposition by atmospheric pressure plasma jet (APPJ). These methods have been also employed to deposit TiO_2_ using APP; among them, the PECVD technique is used most commonly [48]. The wide use of this technique is attributable to its many advantages, primarily its relatively simple and low-cost experimental set-up and its ability to deposit thin films even at low temperatures [49,50,51,52,53,54,55,56,57]. The general process of the PECVD technique to deposit TiO_2_ employs a Ti-based precursor—TTIP is used most often—which is transferred by a carrier gas, typically an inert gas, and then mixed with a feed gas. Thereafter, this mixture is passed through the plasma-generating region (also referred to as the discharge region), where reactive species are formed from the feed gas and precursor. These species are then directed to be deposited on the desired substrate. An example of a PECVD set-up used by Yoshiki et al. is shown in Figure 2 [49]. The authors used TTIP as the Ti precursor carried by He, which was mixed with O_2_ (primary feed gas) before passing to a discharge region [49]. In this method, plasma is generated within a quartz tube between parallel electrodes kept outside the tube and TiO_2_ is deposited on the inner wall of the tube.

In addition to a variety of experimental set-ups, deposition parameters such as the plasma power, flow rate, substrate temperature, and annealing temperature vary in different studies that have used PECVD. Different types of discharges have been also applied. However, most researchers have used either an rf of 13.56 MHz [50,52,53,54,56] or microwave discharges [49,51,55,58,59,60,61] to ignite the plasma. Further, the thickness, morphology, crystallinity, and other properties of the grown films have been diverse in research findings as they depend largely on the deposition parameters. 

Although DBD-based techniques [62,63,64,65] are not as popular as PECVD, they have been used to deposit TiO_2_ in several studies to date. In the DBD technique, a dielectric material is used as a barrier between high-voltage electrodes, which prevents arcing during the plasma discharge at atmospheric pressure. For this type of plasma ignition, most researchers have used a power supply that generates a plasma frequency in the range of kHz rather than MHz. The use of DBD methods for thin film deposition is discussed more extensively in two of Fanelli et al.’s review articles [13,66].

**Figure 3:**
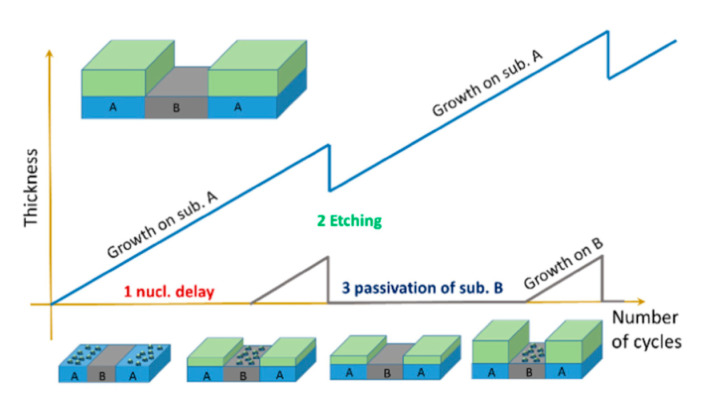
Schematic of an atomic layer selective deposition process as an alternative method for three-dimensional patterning. The supercycle consists of three steps, in which (1) nucleation times on two substrates (A and B) differ due to different surface chemistries on each surface; (2) a precise and selective plasma etching process is used to remove undesired growth on substrate B; and (3) chemical passivation is used to prevent material growth on substrate B. Reprinted with permission from [69]. Copyright (2019), American Vacuum Society.

In the PEALD method, thin films are grown at a very low deposition rate per cycle, which results in a thickness comparable to the atomic layer thickness [67,68]. However, the precursor and plasma gas are supplied intermittently in the form of pulses in PEALD, unlike in other deposition techniques, in which they are supplied simultaneously in a continuous mode. The deposition of films is obtained in cycles, in which each cycle is divided into multiple pulses, beginning with the precursor pulse (i.e., when a precursor is fed) and reactant pulse (i.e., when plasma gas is fed as well as when plasma is generated). The purging gas is then fed during the purge pulse, which is generated after each precursor and reactant pulse. Moreover, TiO_2_ films can be alternated and compositionally controlled by introduction of other materials in a so-called supercycle. In a previous PEALD study, a typical cycle was used to deposit TiO_2_ thin films, while Al-doped TiO_2_ thin films were deposited in a supercycle, which was made up of a number of cycles of Ti precursor followed by one cycle of Al precursor [68]. Recently, a supercycle process was used to selectively deposit TiO_2_ on defined regions over substrates by intercalation of plasma etching cycles in PEALD [69]. In this approach, three steps were performed as is presented in Figure 3.

In another deposition method, Yuji et al. [70] and Ha et al. [71] used a plasma torch to deposit TiO_2_, where the discharge was created between an anode and a cathode that consisted of a Ti rod to produce plasma in the form of a jet. Moreover, Fakhouri et al. [16] and Jimenez et al. [72] used completely different set-ups to introduce the precursor in the post-discharge zone. Fakhouri et al. sprayed the precursor liquid directly into an open-air APPJ, while Jimenez et al. mixed precursor vapor with a carrier gas before introducing the precursor into the post-discharge zone.

Technical aspects of the APP deposition methods largely vary across research groups because these technologies are still in the development stage and the majority of them are based on lab-built instrumentation; thus, they are only briefly described above. 

## 5. Atmospheric Pressure Plasma-Enhanced Deposition of TiO_2_

### 5.1. Plasma-Enhanced Chemical Vapor Deposition

Plasma deposition of TiO_2_ dates back to the 1960s, but only in the early 1980s, Williams and Hess [73] investigated the structural properties of TiO_2_ films grown from TiCl_4_ and O_2_ mixtures and the correlation between crystalline TiO_2_ phases and plasma variables. Their research showed significant phase changes in a deposited TiO_2_ film, from the amorphous to anatase phase and then to rutile, with an increasing post-deposition annealing temperature that ranged from 300 °C to 600 °C. A year later, the authors explored and produced high-quality TiO_2_ photoanodes on a polycrystalline Ti substrate grown at several different rf powers during PECVD [74]. They observed that, as the rf power increased, the relative quantum efficiencies of the films decreased. More importantly, compared to films grown thermally, the research findings revealed higher quantum efficiency below a wavelength of 330 nm. These superior PEC properties were attributed to better crystalline (in this case the rutile TiO_2_ phase) quality of the film and smaller amounts of chlorine incorporated. Thus, this finding indicated that particular chemical reactions attainable only by plasma deposition, not conventional methods, can enhance certain PEC properties of a thin film. 

**Figure 4:**
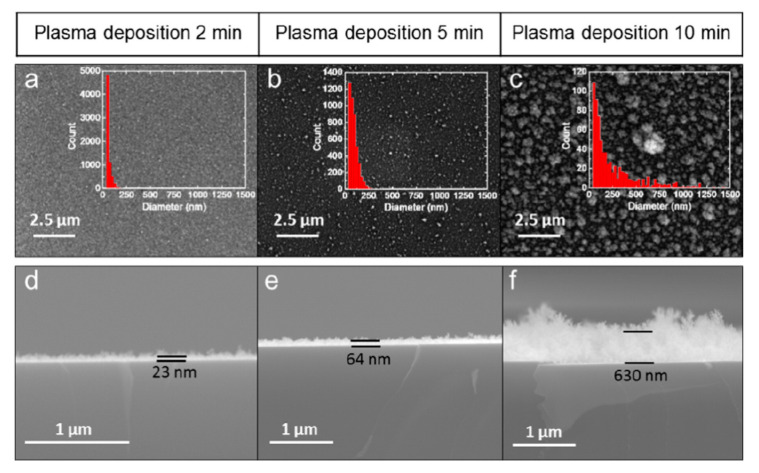
Surface morphology of TiO_2_ films on a Si wafer with increasing deposition time (**a**–**c**). Insets showing histograms of particle size distribution. As the deposition time increases, the film thickness and roughness increase (**d**–**f**). These scanning electron microscopy (SEM) images were reprinted from [75], with permission from Elsevier.

In the early 1990s, to avoid chlorine-related hazards from the TiCl_4_ precursor and to counter chlorine contamination, metalorganic compounds, such as TTIP, TEOT, Ti(O-i-C_3_H_7_)_4_, and Ti(OCH(CH_3_)_2_)_4_, were used to grow TiO_2_ films. Further, these precursors allowed the surface quality to be improved. For example, a mixture of Ar (or other inert gases) and TTIP was used to deposit an amorphous, carbon-free, scratch-resistant TiO_2_ film on Si wafers or glass using PECVD [57]. In this study, the stoichiometry of the deposited TiO_2_ was independent of the gas phase parameters and the substrate temperature, although the film density, which is correlated with the refractive index and stability, varied with the gas phase parameters and substrate temperature during and after deposition. Further, Lee et al. performed a thorough investigation of the structure, chemical composition, and optical and electrical properties of amorphous TiO_2_ deposited on clean Si(100) using TTIP as a precursor, and found that two parameters, i.e., the deposition rate and refractive index of the TiO_2_ thin film, changed with varying rf power, gas pressure, and substrate temperature [56]. However, both parameters remained the same for various oxygen flow rates, although spectroscopic studies confirmed the presence of contamination derived from carbonaceous compounds and water. A depth profile indicated that the films prepared under optimal conditions were homogeneous, with an O:Ti ratio of 2, and that carbonaceous contamination decreased gradually with depth. Moreover, a partially crystalline film could be obtained at lower temperatures when the deposition time was long enough to allow the deposited atoms to be displaced sufficiently to form a crystal. Even so, the longer deposition time increased the surface roughness because of a small crystallite growth. It is worth noting that, previously, the deposition time required several hours [56], while the current plasma methods operate with a substantially reduced deposition time (for example, see Figure 4) [75]. While the TiO_2_ film deposited in Lee et al.’s study exhibited a small capacitance−voltage hysteresis, the dielectric constant increased after the film was treated with N_2_ and O_2_ plasma. Overall, the post-deposition plasma treatment of the film by O_2_ improved the quality of the TiO_2_–Si interface by reducing the oxygen vacancies and defects [56]. Later, in the early half of the 2000s, the advantages of PECVD were reinforced when Maiti et al. investigated TiO_2_ thin films deposited on strained Si–Ge layers and strained Si layers with interfacial traps [55,60]. In both of their works, they used TTIP with oxygen in their microwave discharge system for low-temperature deposition. The x-ray diffraction (XRD) results demonstrated that a longer plasma deposition time led the fully amorphous phase to convert to partially crystalline films at a low substrate temperature. In general, such a conversion was observed either when the substrate temperature increased and/or when the layer deposited was thicker. Both of these factors are known to help form crystal structures. 

Studies published in 2019 compared the effects of rf APP with thermal treatment on crystallization of amorphous TiO_2_ films [76] and also reported the effect of plasma synergy with substrate temperature [77]. The study showed that the plasma improved the crystallization significantly, and the phase transition to the anatase film was observed at a lower temperature and in a shorter time [76]. However, some undesired etching also occurred, during which some material was removed [76].

In PECVD, other factors, such as plasma reactive species (e.g., ions), rather than temperature alone, can contribute to the formation of films with different crystalline morphologies. The correlation between plasma species generated in the discharge and the elemental composition of the deposited films is explored often [78]. One of the most common plasma diagnostic methods to identify plasma species is OES, which detects light emitted from excited plasma species and can provide certain useful information, such as plasma species density and their energy distribution, and help interpret the growth mechanism. The presence of specific ions created in plasma in the PECVD technique promotes precursor dissociation at the surface and thus makes deposition possible at a lower temperature compared to conventional deposition techniques [78]. Since PECVD was introduced, much research has been dedicated to understanding the role of plasma-reactive species in TiO_2_ film growth [12].

For example, in early studies, Lee investigated the role of energetic ions in plasma-enhanced deposition [59] and explained that ions are responsible for both etching and the increased density of the deposited film. Due to the densification of the deposited TiO_2_ film in rf-biased PECVD, the anatase phase dominated over the rutile phase below 400 °C, while the rutile phase became dominant at a higher annealing temperature. An interesting comparison between low-pressure chemical vapor deposition (LP-CVD) and PECVD-grown films revealed that PECVD produces a smoother, denser, and more continuous TiO_2_ layer than does LPCVD [79]. However, in contrast to LPCVD, two effects, i.e., a lower deposition rate and poor crystallinity, were drawbacks of PECVD in this particular study. These effects were attributed to the ion bombardment and high fragmentation of the precursor during the process, which hindered the Ti–O bond formation.

Another study, which investigated the mechanism of TiO_2_ deposition using the PECVD method, revealed that H and OH radicals in an H_2_/O_2_ mixture played a key role in this process [80]. The contribution of both radicals was confirmed by a comparative study in which only an O_2_ gas plasma was used. The deposition rate for the O_2_ gas plasma, in which O radicals were produced, was insignificant compared to that for the H_2_/O_2_ mixture. Moreover, the authors observed that the deposition rate was strongly correlated to the ratio of both radical densities, with the maximum deposition rate reached at 80% H_2_ [80]. Thus, it was concluded that two competitive factors affected the deposition rate and film morphology in low-temperature deposition: dissociation and activation of precursor molecules by H radicals and deactivation of precursor molecules by OH radicals. Although mixing O_2_ in the feed gas enhanced the H radical density in the plasma, a high deposition rate could only be achieved by optimizing the gas phase parameters (i.e., gas composition and flow rate) and plasma power. This study also confirmed previous research findings which emphasized the contribution of H_2_ gas admixture in open-air PECVD used to deposit highly dielectric amorphous films [71]. In the absence of annealing, there was no crystallization in the film grown by PECVD because of the structural defects and vacancies on the surface. Interestingly, the substrate temperature had no significant effect on the deposition rate in this study. However, increasing the substrate temperature decreased the content of OH groups in the film and increased Ti−O−Ti bond formation, thus resulting in a high refractive index and small band gap in the deposited material. Moreover, research has found that efficient fragmentation of precursor molecules on the surface caused lower carbonaceous contamination in PECVD-grown films compared to those grown using LPCVD [81]. Note that the carbon content increased with increasing plasma power. Nonetheless, LPCVD produced a partial anatase phase at a substrate temperature of approximately 350 °C, while the thin film produced by PECVD had to be annealed under ambient pressure at 400 °C to obtain an anatase crystal. This observation indicated that the PECVD film had a considerable number of defects and oxygen vacancies initially, which hindered the crystallinity. However, PECVD film was smoother and highly conformal, exhibiting dense microstructures [82]. 

A further comparison of PECVD and LPCVD showed that the former exhibited a faster deposition rate, which might result from a reduced activation energy barrier, as was mentioned above [54], and a lower O_2_ concentration and rf power. A higher O_2_ concentration led to a high density of atomic oxygen, which then oxidized the precursor molecules before deposition, rendered them inactive, and hence resulted in a low deposition rate. Atmospheric pressure chemical vapor deposition without plasma also led to a much lower deposition rate compared to PECVD when similar experimental set-ups were used for both techniques, and thus makes PECVD attractive for its potential application in industry [83]. A recent study that used APP to coat polymer optical fibers with photocatalytic anatase TiO_2_ thin films showed greater advantages of this method than other commonly used techniques, such as thermal CVD and wet chemical processes in functional coatings [84].

As mentioned above, in studies that used PECVD, precursor dissociation by reactive oxygen led to a very high deposition rate and crystallinity at lower temperatures compared to conventional deposition techniques [85]. However, this method may sometimes lead to less dense grains and, eventually, poor crystallinity in the deposited thin film compared to LPCVD. To improve the crystallinity, Yamauchi and Imai reported the growth of TiO_2_ films in the form of nanosized columnar grains using TTIP in PECVD [52]. The authors also investigated the effect of deposition parameters on the photoinduced hydrophilicity of the deposited film [52], as hydrophilic TiO_2_ can be a very efficient self-cleaning surface [86]. Further, their study showed that the film, which grew at 340 °C, exhibited high hydrophilicity, which increased with increasing temperature up to 380 °C in the absence of UV irradiation and became super-hydrophilic after 5 minutes of UV irradiation [52]. Similarly, super-hydrophilicity and excellent photocatalytic activities were observed for TiO_2_ films grown by post-discharge of rf atmospheric plasmas supplied with oxygen and argon [75].

Another approach to obtain a smooth crystalline TiO_2_ film is to use APP with pulsed injection metalorganic chemical vapor deposition. Jiménez et al. used this method and obtained smooth (i.e., average roughness of 1 nm) titania films on a Si substrate using different deposition parameters [72]. In their study, the deposited film crystalized at 275 °C and higher temperatures. In addition, an increase in deposition temperature removed contamination. Jiménez et al. found that the deposition rate also increased with an increased concentration of Ti precursor in plasma at a particular temperature, but, on the other hand, a lower precursor concentration enhanced the crystallization of the film. Battiston et al. compared the properties of amorphous TiO_2_ deposited with and without oxygen present in nitrogen and argon plasmas [87]. Films prepared in oxygen-free plasmas at low temperatures were smooth, transparent, and highly resistive but had a low conductivity, which increased at higher temperatures. Recently, it was also confirmed that improved transparency to visible light can be achieved when the roughness of TiO_2_ films is reduced [32], while films prepared in the presence of oxygen were rougher and had little optical absorbance but showed high efficiency in photocurrent generation [87].

**Figure 5:**
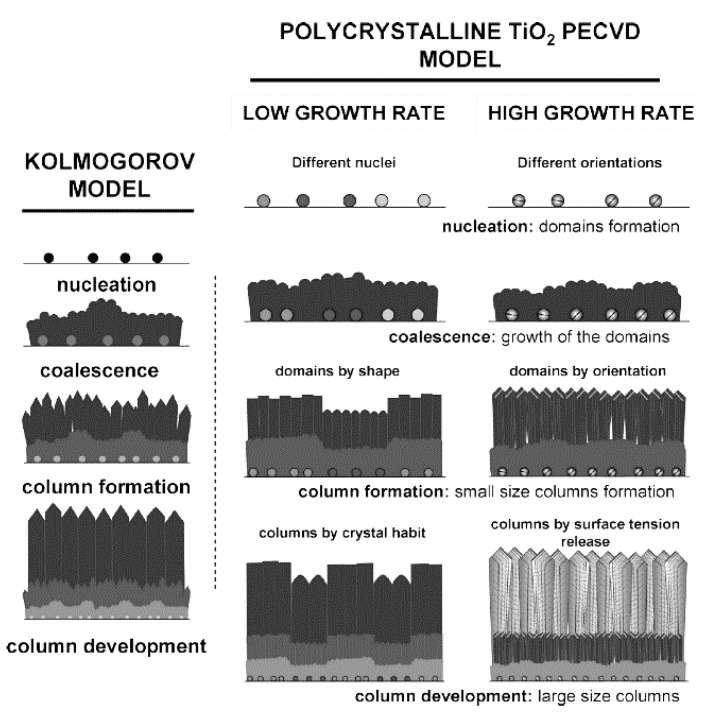
Schematic diagram of a Kolmogorov model (left) and TiO_2_ film grown with low and high deposition (growth) rates (right). Reprinted with permission from Ref. [58]. Copyright (2009) American Chemical Society.

At the same time that important factors involved in the deposition rate and crystallinity of TiO_2_ films have been explored, several film growth models have been proposed. In general, the growth process of TiO_2_ in PECVD follows a Kolmogorov model, which has four stages: nucleation, coalescence, column formation, and column development (see Figure 5). In the case of a slow deposition rate, the film is controlled by the materials supplied by plasma and formed through crystal habits, while, for faster growth, steps and facets are formed that release the tension [58]. A study also revealed an additional feature of anatase crystal growth in PECVD: the formation of microstructure domains that the nucleation stage potentially controls. Experimentally, low-temperature (150 °C) PECVD was used to grow micro-columnar, porous TiO_2_ anatase thin films on Si or glass substrates from TTIP [88]. Such a columnar morphology of thin films is beneficial for photocatalytic applications, particularly for membranes in which a high specific surface area and permeability are required.

**Figure 6:**
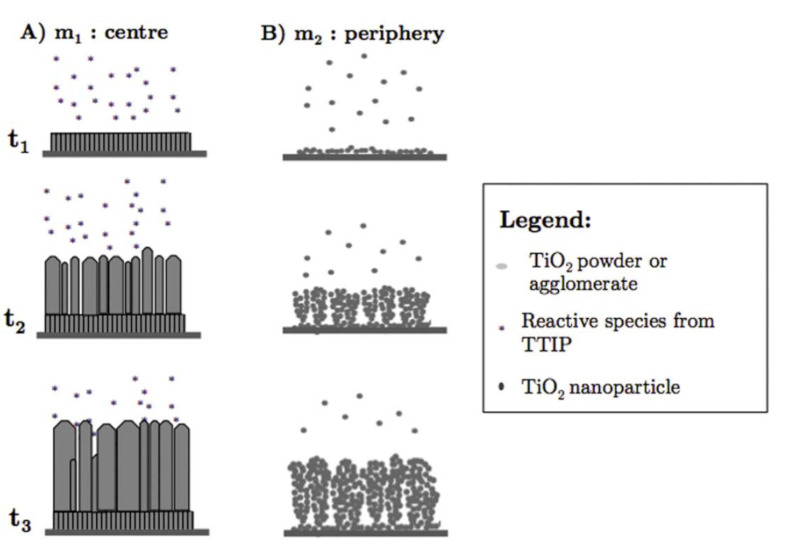
Different growth mechanisms of TiO_2_ films in the (**A**) central and (**B**) peripheral zones during PECVD using a microwave plasma torch. Reprinted with permission from Ref. [51]. Copyright (2016) American Chemical Society.

Gazal et al. proposed another growth model for TiO_2_ deposition with PECVD and a microwave plasma torch [51]. They found that the reactive species differed at the center and circumference of the deposition area because of a temperature gradient from the center to the periphery of the plasma torch. In the central zone of the deposition area, the mechanism of TiO_2_ thin film growth followed the Kolmogorov model, which resulted in a film with a crystalline, columnar structure (see Figure 6A). In the peripheral zone, a cauliflower-like, amorphous structure formed that was a result of the agglomeration of titania nanoparticles formed in the plasma (see Figure 6B). Any temperature changes in these two areas resulted in different diffusion rates, which, in turn, altered the deposition process. The cauliflower-like assembly was observed when PECVD was used to prepare TiO_2_ on various substrates [89,90]. In contrast to studies in which the columnar morphology was observed [88], a plasma jet was incorporated into the experimental set-up in these studies, although both growth processes were carried out at low temperatures. Therefore, the authors concluded that the cauliflower-like structures were grown from deposited TiO_2_ particles produced by the titanium and oxygen reactive species that formed and collided with each other along the plasma jet [89].

An experimental study carried out in 2019 used PECVD combined with APPJ technology to elucidate the potential growth mechanisms of TiO_2_ thin films deposited over areas of several square centimeters [91]. In this study, the substrate’s ability to move allowed the column growth mechanism to be controlled in two modes: static and dynamic. The authors showed that, in contrast to static deposition, the movement of the substrate and a fast precursor flow led to the cauliflower-like morphology, while a low precursor flow promoted surface reactions and led to columnar TiO_2_ anatase thin films [91].

Recently, in parallel to work on the basic principles of film growth, more applied studies have also been carried out that have focused on increasing the photocatalytic efficiency of TiO_2_ films produced using PECVD. For example, Wang et al. revealed that mesoporous material, which is suitable for organic compound adsorption, could be coated with mixed anatase and rutile phases (with a phase ratio of ~55 and 45, respectively) of TiO_2_. This system showed up to 40% photocatalytic efficiency under fluorescent light, which the authors attributed to semiconductor–semiconductor coupling [53]. However, the efficiency under UV light differed. In addition, PECVD was used to coat microporous ceramic supports with nanocrystal anatase films to form composite membranes with photocatalytic activities comparable with those obtained from membranes prepared by the sol-gel process [92]. The authors mentioned that it might be unsuitable to use this method for polymer membranes because of the particular experimental conditions used for this deposition [92], but, later, another group that used a different set-up of APP at low temperature was able to deposit anatase films on polymer optical fiber [84].

Di et al. tested the photocatalytic properties of “as-deposited” and thermally treated TiO_2_ film produced by PECVD at atmospheric pressure [65]. Thermally treated TiO_2_ in air (calcination) exhibited better photocatalytic activity compared to “as-deposited” TiO_2_ by converting formaldehyde into CO_2_ without releasing CO, a toxic by-product. In another study, calcination of hybrid titanium butoxide/polyvinylpyrrolidone fibers was also used to prepare TiO_2_ submicron fibers using diffuse coplanar surface barrier discharge plasma at atmospheric pressure by removing the base polymer and organic part of the precursor [62].

**Figure 7:**
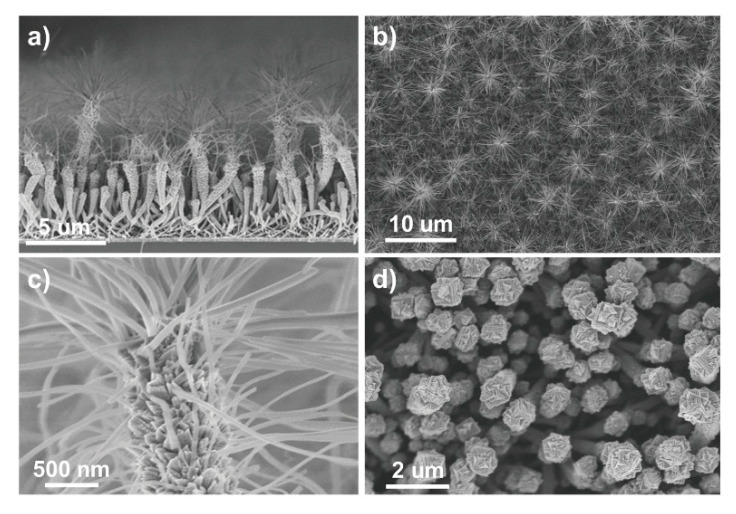
SEM images of TiO_2_ multistacked nanotrees. The images show the different steps in multistacked nanotree formation (**a**–**d**). Used with the permission of the Royal Society of Chemistry, from Ref. [50]; permission conveyed through Copyright Clearance Center, Inc.

Further, TiO_2_ nanotubes were used to prepare photoanodes in DSSCs [50]. In this study, not only was a TiO_2_ thin film deposited, but anatase hierarchical nanotubes and nanotree multistacks were grown on an organic nanowire template using PECVD (see Figure 7). DSSCs fabricated with these nanostructures revealed exceptional efficiency because of the high surface area of the nanostructures, with a much lower film thickness compared to those reported in previous studies [50].

The most important plasma and deposition parameters and post-treatment conditions, and their effects on film morphology, as well as the scope and applications of selected studies using the PECVD technique, are summarized in Table 1. 

### 5.2. Plasma-Enhanced Atomic Layer Deposition

**Figure 8:**
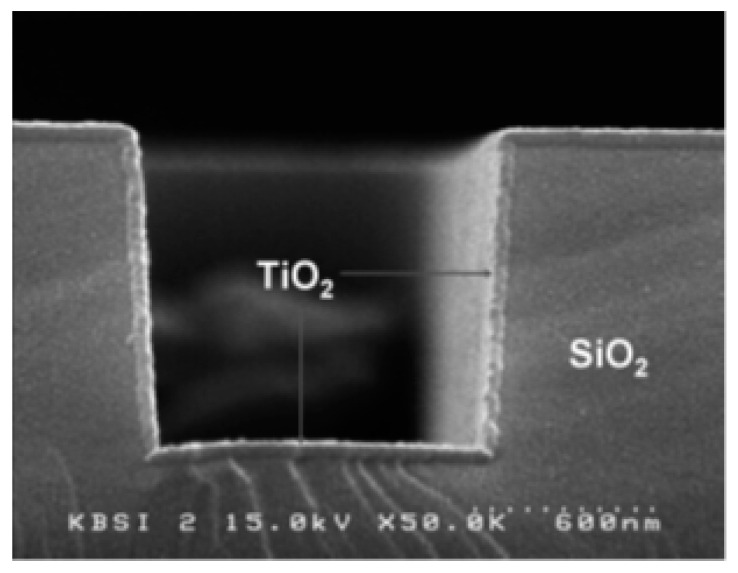
Cross-sectional SEM image of a TiO_2_ thin film obtained using PEALD on a patterned SiO_2_/Si surface maintained at 200 °C. Reprinted from Park et al. [67]. Copyright © 2004 Taylor & Francis Group.

PECVD, as described above, is an excellent technique when fast, low-temperature deposition is necessary. However, it has its limitations when electronic-grade and precisely controlled thin film is required because it is challenging to achieve accurate control with this method. The principal difficulty is multilayer diffusion, as this phenomenon degrades the quality of electronic devices. Therefore, low-temperature, plasma-enhanced ALD (also called plasma-assisted ALD) can be beneficial because multilayer diffusion can be avoided with this technique. In one of the earlier studies, Park et al. deposited a very thin, relatively smooth TiO_2_ film on Si using PEALD [67]. In their approach, they used a Ti(N(CH_3_)_2_)_4_ and O_2_ plasma and obtained precise thickness control and a highly uniform film with insignificant carbonaceous contamination (see Figure 8) [67]. 

Similarly, as in the case of PECVD, ongoing research focuses on exploring such important factors as temperature, gas flow, precursor, bias voltage, and plasma power involved in deposition rate and crystallinity of TiO_2_ films during PEALD, as well as other film properties [95,96,97,98]. 

For example, it has been shown that a substrate bias played a significant role in deriving a specific phase of PEALD-deposited thin films. Profijt et al.’s work, in which the authors were able to alter the TiO_2_ thin film phase property by changing the substrate bias voltage explained this in detail first [95]. In their work, anatase TiO_2_ was obtained at 300 °C without any bias voltage, while the deposited film began to exhibit rutile features in XRD patterns as the negative substrate bias voltage increased (~−100 V). At −200 V, the deposited film exhibited only rutile peaks in the XRD results, which indicated that the crystallization of the film can be controlled by changing the bias voltage rather than the substrate temperature. However, the study did not report other properties of the deposited film with increasing bias voltage at room temperature, such as electrical, optical, or PEC properties, which are essential for future applications of this method. 

On the other hand, studies on the electrical properties of Pt/TiO_2_/Si and Pt/TiO_2_/Pt systems fabricated using PEALD revealed that the dielectric constant of the film decreased slightly with increasing temperature [67]. Moreover, the leakage current decreased when the sample was annealed in an O_2_ environment, which improved the interface quality by reducing the localized charges and trap states at the injection interface [55,60]. However, more investigations of TiO_2_ films produced by PEALD are still necessary.

More recently, Ratzsch et al. showed that varying plasma conditions, i.e., gas flow rate and plasma power, can control the growth rate, refractive index, and surface morphology of deposited TiO_2_ layers from amorphous to crystalline in the anatase phase [96]. The authors found that the anatase phase on the Si substrate was formed at temperatures as low as 70 °C under low oxygen gas flow rates and high plasma power. There was a much higher number of ions, radicals, and electrons under these conditions, which contributed to surface bombardment and reactions that influenced the morphology [96], confirming the findings of a previous study that stressed the importance of plasma species in determining the surface chemistry [99]. Moreover, as two independent studies have shown [96,97], the growth ratio increased with plasma power when either TTIP or TiCl_4_ was used as a precursor [97] but decreased with plasma power greater than 100 W for TTIP. 

Further, in comparison with the deposition rate of PECVD, conventional ALD is a slow deposition process, which makes it inefficient for large-scale production. Moreover, doping a thin film at the time it is deposited can cause the ALD process to slow even further. On the other hand, PEALD exhibits a significantly higher TiO_2_ deposition rate, at lower temperatures (150 °C), than does conventional ALD, as was reported for O_2_-PEALD in comparison to ALD of TiO_2_ using water and ozone [100]. Later, Theirich et al. demonstrated the APP ALD of TiO_x_ layers at room temperature for the first time using O_2_/Ar flow [101]. The authors concluded that the metastable excited argon atoms are essential in the TTIP precursor decomposition because the growth was independent of the presence of oxygen. This study also showed that the deposition rate by room-temperature PEALD was at least twice as high than with thermally activated ALD, which can be more beneficial for industrial roll-to-roll manufacturing [101]. Later studies confirmed room-temperature PEALD of amorphous TiO_2_ films, and also showed the formation of the anatase phase at temperatures over 60 °C and 180 °C using O_2_/Ar and H_2_O/Ar plasmas, respectively [98]. 

Extensive research has been performed to determine whether it is possible to increase the deposition rate of rutile TiO_2_ grown on Ru using PEALD [68]. In this work, the researchers used O_2_ or N_2_O plasmas and investigated effects on the deposition rate, structure, and electrical properties of both pure TiO_2_ and Al-doped TiO_2_ films. The results showed that N_2_O plasma increased the deposition rate by nearly 80% compared to O_2_ plasma by producing active oxygen atoms more efficiently during the PEALD process [68]. This effect was attributed to the weaker adsorption energy of N_2_O compared to O_2_. The study also revealed that nitrogen was adsorbed after the N_2_O plasma pulse and was then removed during the subsequent precursor pulse and purge steps. Overall, the final film contained no nitrogen species. It was concluded that N¯ ions contributed to ligand removal during the precursor pulse step in addition to the reactions promoted by the reactive oxygen atoms [68]. Not only the deposition rate, but also film density, was higher for N_2_O plasma in this study because of the greater amount of O¯ ions, which were accelerated to the substrate because of the self-bias effect. The negative ions impinged on the growing film surface physically and resulted in much denser films. It is also important to note that an RuO_2_ interfacial layer with a rutile crystal structure was formed in this study during plasma deposition of TiO_2_ and, therefore, caused the formation of rutile TiO_2_ to be more favorable at low temperature. In the case of N_2_O plasma, the RuO_2_ layer was denser because of active O¯ ions, which led to complete rutile TiO_2_ formation. Despite the structural and compositional changes using O_2_ or N_2_O plasmas, the leakage current at a particular thickness of the film grown by N_2_O was similar to that of the film grown by O_2_ plasma. In conclusion, this study was the first step toward the development of a plasma deposition process for electronic-grade TiO_2_ films with the potential for industrial scale-up for mass production. 

In addition to exploring the direct effect of ions and excited species on TiO_2_ film growth, two different plasma modes, i.e., low and high electron current modes, which are characterized by different electron temperatures (lower and higher, respectively) [102], were investigated recently [103]. In the case of the low-current mode, the films obtained were deposited across the substrate areas uniformly. In this mode, the most dominant plasma species observed by OES were atomic oxygen radicals, formed primarily via the dissociative electron attachment process. In contrast, non-uniform film growth was observed with the high-current mode. This observation was attributed to electron avalanches that developed because of the secondary electrons from the substrate [103]. Thus, this mode is not applicable for general PEALD use. 

Because it is still challenging to use PEALD to manufacture TiO_2_ thin films roll-to-roll, other possibilities to use this technique for industrial applications were explored. Very recently, a micro-plasma printer was employed to deposit TiO_2_ thin film with desired patterns using PEALD at atmospheric pressure with a N_2_/O_2_ gas mixture [93]. The properties of TiO_2_ films deposited by this method showed a higher refractive index and lower level of impurities compared to the properties of films deposited using the CVD technique. Moreover, the patterning resolution was twice as high as those from the latter technique, but it still requires more improvements to be applied in plasma printing technology in area-selective deposition [93]. 

PEALD is a deposition technique widely used today to grow ultra-thin films of various materials other than TiO_2_, and the benefits and challenges associated with this technique have been addressed in two review articles published in 2011 [104] and 2019 [105]. Niemelä et al. published another extensive review dedicated solely to TiO_2_ thin films deposited using ALD techniques, including PEALD [106].

The most important plasma and deposition parameters and post-treatment conditions, and their effects on film morphology, as well as the scope and applications of selected studies using PEALD technique, are summarized in Table 2. 

### 5.3. Atmospheric Pressure Dielectric Barrier Discharge Deposition

Finally, the last method for TiO_2_ plasma deposition described in this review is APP ignited based on DBD, including atmospheric filamentary discharge [107], diffuse coplanar discharge [108], and APPJ, for which the term “torch” is used sometimes rather than “jet”. Moreover, a combination of two methods, e.g., APPJ with CVD [90,91,109], can be used to synthesize and/or treat materials. 

In 2018, atmospheric filamentary DBD was used for the first time to synthesize TiO_2_ films with a different morphology that can be achieved in a controlled manner [107]. In this work, the effect of various plasma parameters, i.e., gas flow rate, concentration of TTIP precursor, total gas flow, plasma power, and deposition time, was investigated systematically. By tuning these parameters, the density of the deposited films varied from loosely dense powders to a dense and compact anatase crystalline phase with enhanced photocatalytic activity [107]. Moreover, the authors proposed a deposition mechanism that explained the effect of plasma parameters on the film morphology. This mechanism consisted of three stages, including the formation of excited and reactive species in the discharge, chemical reactions that initiate the nucleation process, and growth saturation processes. According to this mechanism, the morphology of the deposited film depends strongly on the energy density in these stages. This study was extended subsequently to N-doped TiO_2_ films using the same experimental set-up for APP with varying concentrations of NH_3_ in Ar plasma, which led to the formation of NH radicals [43]. A comparison of N-doped films revealed a lower band gap and a higher (approximately 1.4 times) photocatalytic degradation rate constant than those for the undoped TiO_2_ films. 

Similarly, as in PECVD, the APDBDD methodology was also employed in TiO_2_ nanoparticle synthesis with controllable size, distribution, and morphology by tuning such plasma parameters as plasma power, frequency, and the molar ratio of precursors and feed gas [110]. The results from this method were compared with those from the furnace reactor and commercial aerosol generators and showed its great potential as an aerosol generator to produce solid spherical particles. 

In addition to using APDBDD to synthesize nanoparticles, this method has also been used to deposit pre-synthesized TiO_2_ nanoparticles in the form of a nanocomposite thin film onto a Si substrate [111]. This study reported that the advantage of this method was its ability to deposit the nanoparticles directly onto a substrate. Moreover, the discharge parameters, particularly the voltage waveform and frequency, controlled the nanoparticle coverage and thickness. This process is particularly beneficial for industrial scale-up, where film thickness needs to be homogeneous over a large surface area of the substrate. 

Further, APPJ can be used to obtain highly porous films or nanoparticles, which are important for certain applications, particularly when a high surface-to-volume ratio is desired. Although PECVD provides a high deposition rate for thin uniform films, compared to that using APPJ, porosity is very difficult to achieve with the PECVD process.

**Figure 9:**
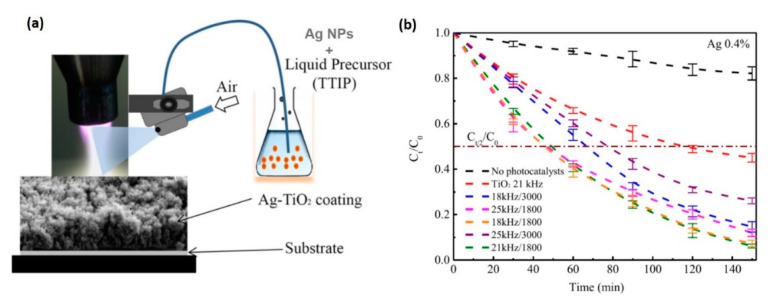
(**a**) Schematic of the APPJ system coupled with a homemade spraying system for one-step deposition of nano-Ag–TiO_2_ coatings from the injection of Ag nanoparticles (NPs, with an average particle size of 100 nm) dispersed in TTIP liquid precursor on two different substrates, glass or Si(100), at 200 °C. All the coatings were annealed in ambient air for 1 hr at 450 °C. (**b**) Photocatalytic degradation kinetics for rhodamine B over 2.5 h on glass coated with the Ag–TiO_2_ catalyst under a 125-W Philips white light source at a fixed concentration of Ag NPs (0.4%) and under different pulse frequencies (kHz) and air flow rates (L/h), compared with the substrate without any photocatalyst, and for the TiO_2_ coating. In particular, the samples prepared with a pulse frequency of 18 or 21 kHz and an air flow rate of 1800 L/h showed the best photocatalytic activities, with a half-life of ~50 min, by which time the number of Rh.Bs decreased by half compared (C_t/2_) to the initial concentration (C_0_ = 5 mg/L). Copyright 2019 Wiley. Used with permission from [112].

In Fakhouri et al.’s recent work [16], a precisely controlled and porous TiO_2_ film grown on membranes using APPJ was compared with a nonporous thin film deposited by rf magnetron sputtering with respect to PEC and photocatalytic activities. On the one hand, a thin film grown by APPJ showed better photocatalytic activity, inducing rhodamine B (Rh.B) dye (i.e., a fluorescent material) degradation more effectively in the presence of TiO_2_ with elevated porosity under white light irradiation than in the sputtered deposited film. On the other hand, the PEC responses were superior for rf magnetron-sputtered deposited film [16]. Similarly, Rh.B was used to investigate the photocatalytic activity of nano-Ag–TiO_2_ coatings deposited by APPJ (Figure 9) [112]. By combining the APPJ with a spraying system, high deposition rates were achieved in the range of 10–20 μm/s. Using this method, large photocatalytic activities were measured for coating from solutions with 0.3% and 0.4% Ag concentrations in TTIP. In this study, the authors concluded that pulse frequencies had a negligible effect on Rh.B degradation, whereas lower air flow rates induced a faster kinetics because such conditions caused higher crystallinity of the coating [112]. 

In general, the high porosity of APPJ-grown films was attributed to the high deposition rate and high kinetic energy of the reactive plasma species (i.e., radicals) generated and carried in the plasma jet. In this process, the plasma species on the surface have a limited time to diffuse, which promotes higher porosity [16]. The high kinetic energy of plasma species, which is another contributing factor to the high porosity, was also responsible for the lower surface mobility of these species. In addition to investigating the deposition rate, the crystalline phases of APPJ-deposited TiO_2_ films were explored [16]. This research showed that the anatase crystalline phase was dominant in the case of films with a highly porous morphology. Moreover, regardless of the deposition parameters, the crystal size increased with increasing substrate temperature as well as increasing pulse frequency, which consequently increased the temperature. It is worth noting that carbonaceous contamination of the film quenched with increasing frequency. 

Several other investigations that used atmospheric DBD focused on finding optimal conditions for TiO_2_ film deposition that exhibited high photocatalytic activity. For example, Boscher et al. used an atmospheric pressure N_2_ blown arc discharge with an injection of TIPO as a precursor to deposit anatase TiO_2_ thin films, which were then tested for their photocatalytic activity [94]. In this study, key factors, such as power density, the nozzle-to-substrate distance, and the injector-to-substrate distance, were varied to identify the optimal operating conditions. The results showed that reducing both distances led to a shorter residence time and a more reactive zone between the injector and the substrate. These conditions contributed to the formation of anatase crystalline clusters, which improved the photocatalytic activity of films [94].

In another study in which TiO_2_ was also used for energy conversion-related applications, a mesoporous TiO_2_ film was fabricated on a small area via an APPJ method using TiCl_4_/O_2_ precursor [64]. The results showed that the deposition rate was 25 times higher when this method was used than the ratios reported using PECVD. Some chlorine-based and carbonaceous contamination was present in the “as-deposited” film, but this could be removed by post-deposition annealing. Nevertheless, the films deposited with this method exhibited an almost 50% higher photoconversion efficiency, attributed to high porosity compared to commercial TiO_2_ nanoparticles, when both were employed as photoanodes in DSSCs [64]. Further, fluorine-doped tin oxide (FTO)-coated glass was used as the substrate in this study, because this material favored the formation of anatase TiO_2_ at 200 °C. Similarly, TiO_2_ thin films deposited on a FTO substrate were treated by Ar/N_2_ APPJ to dope the surface with nitrogen atoms [70]. The anatase crystalline phase increased because of plasma treatment, which was then used for DSSC assembly. Overall, this treatment reduced any impurities and incorporated nitrogen atoms on the surface, which enhanced DSSC’s performance by approximately 1.5%. The studies above have shown that post-deposition treatment of thin films by APPJs was beneficial in these cases, particularly those in which chemical composition and intrinsic thin film properties were changed in a controlled manner. Another advantage of APPJ (as well as diffuse coplanar DBD [108]) is to use plasma for sintering in DSSC applications [22,113,114]. Several studies that have used various analyses of TiO_2_ films and nanoparticles demonstrated plasma’s ability to reduce time and temperature in material sintering significantly in comparison to thermal sintering. 

In another study, Chou et al. also showed the benefits of APPJ treatment used to calcinate a TiO_2_ photoanode incorporated into a DSSC [115]. The authors were able to obtain a significantly shorter time for calcination of electrodes for the DSSC, which exhibited cell performance comparable to that obtained from a cell for which a furnace was used for electrode calcination. APPJ’s ultrafast deposition and calcination of photoanodes enables the rapid preparation of DSSCs, which makes this technology suitable for industrial roll-to-roll fabrication [115]. A recent review summarized a large number of studies and indicated future directions for the technology, in which APPJ can be used to process porous materials, including TiO_2_, to advance its applications in energy harvesting and storage devices [116]. 

In addition to using APPJ to form porous films, this methodology was also used to produce TiO_2_ powders from a TiCl_4_ precursor and air [117]. In this study, the morphology varied when the distance between the plasma nozzle and the substrate was changed, demonstrating the appearance of the anatase crystalline size for powders deposited at short distances when the gas temperature was the highest, while, at greater distances, the size of powder granules and Cl contamination increased [117]. 

The use of APPJ based on DBD to coat and treat materials has been a rapidly growing methodology over the past two decades. Because of its undeniable industrial advantages, such as the reduced cost of instrumentation, lower operating temperatures, easier handling and maintenance, convenient operation, and possibility of combining it with other methods, it is an appealing tool as an alternative deposition technique. Its drawbacks are the greater number of plasma parameters that need to be tuned to obtain the desired effect, as well as the possibility of material/surface contamination and more complex physical and chemical processes in plasma, as APPJs are often operated in open air. A recent review paper provided a critical view of APPJ technology in surface processing of materials, including TiO_2_ [66].

The most important plasma and deposition parameters and post-treatment conditions and their effects on film morphology, as well as the scope and applications of selected studies using different types of atmospheric pressure DBD-based discharge deposition technique, are summarized in Table 3.

## 6. Summary and Outlook

This review reported three common APP-enhanced methods to deposit TiO_2_ thin films: plasma-enhanced chemical deposition, atomic layer deposition, and atmospheric pressure–dielectric barrier discharge deposition. In most of the works reported in the literature, the TiO_2_ thin film was deposited using the plasma-enhanced chemical deposition method because of its simplicity, low cost, rapid deposition, and low-temperature regime. Other methods were also used, but they were explored for specific applications, such as photocatalytic and photo-electrochemical processes.

This review also summarized recent findings on the effects of various deposition parameters on the structural, electronic, and optical properties of TiO_2_ films using these three methods. An excellent benefit of these methods is that, by tuning the deposition parameters, one can engineer TiO_2_ films that can then be exploited for a range of applications, particularly energy conversion devices. 

One of the focuses here was on comparing the deposition rates using these different methods. As is now well established, PECVD and APPJ can be used for much faster deposition than conventional deposition methods, while PEALD can be used for slow and more controlled layer-by-layer deposition. However, researchers have recently shown that PEALD can also be useful for commercial production.

Further, the formation of the final phase of TiO_2_ thin films or nanostructures, as well as the transition between phases, was another particular focus in this review. For example, amorphous smooth films can be obtained at room temperature using PECVD, while the anatase phase was found only when a substrate was at an elevated temperature during growth or post-deposition annealing (between 200–300 °C). Further, the rutile phase is obtained at an even higher temperature. However, the latest studies have also shown the formation of TiO_2_ anatase films using room-temperature PEALD. 

Nevertheless, conventional deposition techniques need a higher temperature to achieve crystallinity than the plasma-enhanced methods. This is because reactive species produced in the plasma discharge can help produce crystalline film. This is a significant advantage in certain specific applications in which high temperatures could lead to undesired material alteration. 

Moreover, because the crystal phase influences film properties—for example, the anatase phase exhibits a lower dielectric coefficient—the anatase/rutile mixture ratio can be controlled by changing the plasma reactive species energy. In the case when ions and electrons possess excessively high energy, they can hinder film growth and crystallization by preventing the densification and creating defects and vacancies. In contrast, post-deposition plasma treatment can produce better crystallinity by removing the oxygen vacancies, which increases the photocatalytic efficiency of TiO_2_ films.

In addition to deposition rate and crystal phase transition, the possibility of impurity incorporation was also addressed in this review when the results obtained with a particular deposition method were described. 

Although TiO_2_ deposition by APPs has been studied and examined in detail, there are numerous unexplored areas that remain to be addressed. More research on consistency is needed with respect to the replicability of crystal phases, which requires a complete understanding of fundamental processes, including plasma reactive species interactions with the surface. Understanding such surface reactions will provide further opportunities to control the structural, electric, and optical properties, which several factors can influence. Among these, deposition parameters, e.g., flow rate of plasma gases and precursors, choice of precursors, plasma power, substrate temperature, deposition duration, and so on, as well as their synergistic interactions, are crucial in altering films. In addition to empirical effort toward improving the description of the effects of the plasma parameters on the attributes of deposited films and their evolution, theoretical and computational efforts are required to deepen our understanding and fill in the knowledge gaps that are challenging and more time-consuming, and thus, more expensive to investigate experimentally. Therefore, predictive modeling of various features of deposited films for any plasma parameter can provide immense opportunities to shed light on this challenge. Nowadays, predictive modeling with machine learning is emerging as a ubiquitous technology, and its scope and application to material science and specifically to material design is receiving significant research attention. There are reported works on using machine learning algorithms to model plasma surface interactions occurring in a given target [118,119,120]. Employing machine-learning-based models not only helps to predict a given outcome, but it also opens possibilities to find the optimal and desired parameter combinations that would be ideal for the deposition process. The scope of implementing and blending predictive modeling, specifically with modern machine learning algorithms, provides great prospects and should be considered to explore the APP field further. Moreover, in order to apply machine learning algorithms, a large, high-quality and reproducible data set from experimental studies is needed. However, impurities and defects can be either beneficial or disadvantageous, depending on the applications of the synthesized materials. Hence, more insight is required into plasma deposition techniques that can be used to control impurities and defects. Although the plasma-enhanced deposition rate is typically higher than that of other conventional techniques, it is still far from industrial scale-up and replicability of the electronic grade of TiO_2_ thin films. Synthesizing different nanostructures, deposition of pre-synthesized nanoparticles of TiO_2_ by plasma, and plasma treatment also face similar challenges. 

Research on APP deposition has immense potential to be one of the most popular research topics in the coming decades. Overall, APP deposition can compete with the conventional deposition techniques used in scientific laboratories as well as in industrial research. 

## Figures and Tables

**Table 1:** Summary of deposition and plasma parameters, post-deposition treatment conditions, and their effects on TiO_2_ film morphology for PECVD. Studies are ordered in terms of decreasing deposition rates.

Precursor	Carrier Gas (Flow Rate)	Feed Gas (Flow Rate)	Plasma Power ^f^	Frequency	Substrate ^c^ (Temperature)	Deposition Rate, nm/min	Film Crystallinity (Conditions, Crystallite Size)/Morphology (Conditions, Particle Size)	PD Treatment (T_PD_, Time) ^d^	Effect of PD Treatment	Scope of Study/Applications	Year [Ref]
TTIP	N_2_ (glass: 25 SCCM, Si: 53 SCCM)	O_2_ (glass: 0 SCCM, Si: 0–10 SCCM)	1–9 kV	60–70 kHz	silicate glass; Si ^b^	glass: 4700–5500 Si: 3250	Amorphous/rough with particle-like features, macroporous (glass); smooth (Si)	-----	-----	Fabrication; Optics	2019 [93]
TiCl_4_	Ar (25 SCCM)	Ar (1000 SCCM) + O_2_ (10 SCCM)	11 kV	10 kHz	quartz (no heating or 100 °C–400 °C)	1000–3000	Amorphous (no heating); anatase (T_sub_ ≥ 300 °C)/dense and fluffy (≤200 °C); dense cauliflower-like aggregates (≥300 °C)	Annealed in Ar (275 °C, 2 hrs) ^e^	No change in crystallinity	Photocatalysis	2019 [77]
TEOT	He (20 SCCM)	He (300 SCCM) + H_2_ (0–25 SCCM)	105 W	13.56 MHz	Si (200°C–500 °C)	900 (T_sub_ = 500 °C)	Amorphous (T_subs_ < 350 °C), Crystalline (anatase phase, >350 °C)	----	----	Synthesis; Electronics	1996 [71]
TiCl_4_ + methanol/ethanol/propanol/H_2_O	N_2_ (0.1–0.4 dm^3^/min)	N_2_ (7.1–11 dm^3^/min)	-----	-----	SiCO coated glass (500°C− 650 °C)	300	Crystalline anatase phase (40 nm)/uniform	----	----	Photocatalysis; Hydrophilicity	2002 [20]
TTIP	Ar (P_Ar + TTIP_ = 15.5–22.5 Pa) ^g^	O_2_ (P_O_2__ = 17.7 Pa) ^g^	50 W	-----	Si; anatase seeded Si; quartz (150 °C)	50–300	Amorphous/micro-columnar, porous	Annealed in air (300 °C, 5 hrs)	Crystalization (anatase, 20 nm)	Photocatalysis; Hydrophilicity	2015 [88]
TiCl_4_	-----	O_2_ (0.9 SCCM)	10 W	13.56 MHz	Glass; Si; Ti foil; sapphire; NaCl plate (25–700 °C)	10–300	Glass: amorphous (T_sub_ < 300 °C); anatase (up to 400 °C; rutile & anatase (500 °C);rutile (600 °C)/Si: smooth (200 °C), crystallite (≥400 °C)	----	----	Synthesis; Plasma diagnostics	1983 [73]
TEOT ^a^ (10 µL/min)	Ar; Ar/O_2_: 80%/20–0%/100% (1 SLM)	Ar (10 SLM)	200 W	2.45 GHz	polymer	75	Crystalline anatase (19 nm)/homogeneous with spherical particles (30–150 nm)	----	----	Photocatalysis	2017 [84]
TTIP ^a^	N_2_ (10 SCCM)	O_2_ (2 SCCM)	20–50 W	13.56 MHz	Si (240 °C–420 °C)	58 (T_sub_ = 320 °C)	Amorphous (Ts_ub_ < 240 °C); crystalline anatase ( >240 °C)	-----	-----	Plasma diagnostics; Optical films	2003 [85]
TTIP ^a^	Ar (0.2–0.5 L/min)	Ar (5 L/min)	6 kV	50 Hz	Si; quartz (25°C–90 °C)	44	Amorphous/rough, granular	-----	-----	Coatings	2018 [32]
TTIP	Ar	H_2_ (70) + N_2_ (0–35) + Ar (20 SCCM)	58–73 W	1.548 kHz	Steel	40	Amorphous/smooth, dense and columnar surface	Annealed in air (400 °C)	Crystalization (anatase)	Hydrophilicity; Mechanics	2006 [81]
TTIP	N_2_ or Ar	N_2_ or Ar (15–20) + O_2_ (0–5 SCCM)	100–500 W	13.56 MHz	Glass, ITO (120 °C–250 °C)	10–25 (N_2_); 17–37 (N_2_ + O_2_ plasma)	Amorphous/smooth (N_2_, T_sub_ = 120 °C); rough and granular (N_2_, 250 °C or N_2_ + O_2_ plasma)	-----	-----	PEC cells	2000 [87]
TTIP	Ar (30 SCCM)	O_2_ (15–30 SCCM)	5–20 W	-----	Si (150 °C–450 °C)	3–30	Amorphous (T_sub_ < 450 °C)	-----	-----	Electronics	2006 [54]
TTIP	-----	O_2_	1400 W	2.45 GHz	Strained Si/SiGe	24	-----	-----	-----	Microelectronics	2004 [60]
TTIP	-----	O_2_	1400 W	2.45 GHz	strained SiGe/Si	24	Amorphous (5 min deposition), partially crystalline (anatase, 12 min deposition)	-----	-----	Microelectronics	2003 [55]
TiCl_4_	Ar (14 SCCM)	O_2_ (9 SCCM) + Ar (900 SCCM)	4.2 W	15 kHz	Glass	22	Amorphous	Annealed in air (350 °C–450 °C)	Crystalization (anatase)	Photocatalysis	2009 [65]
TTIP	Ar	Ar (150 SCCM)	65 W	-----	Si (150 °C–310 °C)	15	Amorphous/Homogeneous	Annealed in N_2_ (400–900 °C)	Anatase: 400 °C, rutile: 700 °C; n↗ and d_film_↘ ^h^	Optical and electrical films	1991 [57]
TTIP	He (480–500 SCCM)	O_2_ (0–20 SCCM)	14 W	13.56 MHz	Quartz	13 (He + O_2_, 2% of O_2_	Amorphous: rough surface with craters and wrinkles only He; smooth and transparent (2% of O_2_; micro-nanoparticles (0.3–3 µm) 4% of O_2_	-----	-----	Synthesis	2008 [49]
TTIP ^a^	He (5 SCCM)	H_2_ (48)+ O_2_ (12) or H_2_ (60) or O_2_ (60 SCCM)	500 W	2.45 GHz	Si; quartz (100 °C)	0.16/0.19/10.8: O_2_/H_2_/H_2_ + O_2_ plasma	-----	-----	-----	Optical films; Plasma diagnostics	2001 [80]
TTIP	-----	O_2_	10 W	13.56 MHz	Quartz (150 °C–400 °C)	≈9	Crystalline anatase (T_sub_ > 300 °C)/dense plate-like nanograins (45 nm)	-----	-----	Hydrophilicity	2013 [52]
TOAA ^i^; TIPO ^a^	N_2_ (80%) + O_2_ (20%) (3.5–5 L/min)	N_2_ (80%) + O_2_ (20%) (15 L/min)	600 W	100 kHz	Si (200 °C–300 °C)	6.7	Amorphous (T_sub_ < 270 °C), crystalline (anatase, T_sub_ > 270 °C)/smooth, granular (20 nm)	-----	-----	Low-temperature synthesis	2008 [72]
TTIP	O_2_	Ar (90%) + O_2_ (10%)	400 W	-----	Si, quartz (250 °C)	1.9–5	Crystalline (dominantly anatase)/microstructural domains	-----	-----	Crystal growth model	2009 [58]
TTIP	Ar (80 SCCM)	O_2_ (2–16 SCCM)	20–150 W	13.56 MHz	Si (100 °C–400 °C)	0.916 (T_sub_ = 250 °C, 100 W, O_2_ = 8 SCCM)	Amorphous/smooth (1 hr treatment); partially crystalline/coarse (treatment time ≥ 3 hrs)	N_2_/O_2_ plasma (150 W; T_sub_ = 250 °C; 30 mins)	Increased dielectric constant	Electronics	1994 [56]
TTIP	N_2_ (50 SCCM)	O_2_ (100 SCCM)	100 W	13.56 MHz	Si (450 °C)	0.33/0.63 (30/60 min treatment)	Amorphous/sparse grains (30 min treatment), smooth surface (60 min treatment)	-----	-----	Synthesis process	2002 [79]
TTIP	He (1 SLM)	He (9 SLM) + O_2_ (0 or 0.005 SLM)	70 W	13.56 MHz	Si (dynamic: 5 mm/s)	2.2 nm/pass ^k^	Amorphous/smooth with uniformly dispersed nanoagglomerates (He plasma); smooth, crack-free and smaller agglomerates (He + O_2_ plasma)	Annealed in air (450 °C, 2 hrs)	Reduced carbon content and film thickness	Plasma diagnostics	2018 [83]
TTIP	Ar (1 SLM)	Ar (18 SLM)	370 W	2.45 GHz	Si	-----	Crystalline (center and intermediate), amorphous (periphery)/smooth, porous, columnar grains (center) rough and cylindrical grains (intermediate), cauliflower-like agglomerate (periphery)	-----	-----	Synthesis; Crystal growth mechanism	2016 [51]
TiCl_4_	-----	O_2_	10–200 W	13.56 MHz	Ti foil (200 °C–600 °C)	-----	Crystalline rutile phase (T_sub_ = 600 °C, O_2_:TiCl_4_ = 9:1, 50–300 nm)	-----	-----	PEC cells	1984 [74]
TTIP	-----	-----	400 W	2.45 GHz	FTO (250 °C)	-----	Crystalline anatase/hierarchical nanotube and multistacked nanotrees	-----	-----	Nanoarchitecture; Photovoltaics	2017 [50]
TTIP	N_2_ (0–50 mL/min)	O_2_ (50–100 mL/min)	0–150 W	13.56 MHz	MCM-41/Silica (200 °C–500 °C)	-----	Amorphous	Annealed (0–800 °C)	Crystalization (anatase + rutile; particle size: 30.3–60 nm	Photocatalysis	2012 [53]
TTIP	Ar (0.7 SLM)	Ar (17 SLM)	420 W	2.45 GHz	Si	-----	Crystalline anatase (dominant)+ rutile/columnar, cauliflower-like structure composed of nanoparticles (10–20 nm)	-----	-----	Synthesis	2019 [91]
TTIP	Ar (0.2 SLM)	Ar (17 SLM)	420 W	2.45 GHz	Si, FTO	-----	Si: crystalline anatase/columnar, faceted grains with intercolumnar porosity; FTO: amorphous/columnar, cauliflower-like assembly	Ultrasound cleaning	Improved optical transmission of films	Synthesis; Solar cells	2019 [89]
TTIP	Ar (P_Ar+TTIP_ = 0.225 mbar) ^g^	O_2_ (partial pressure, 0.177 mbar) ^g^	50 W	-----	Si; porous (100 nm; 800 nm) Al_2_O_3_ (150 °C)	-----	Amorphous/micro-columnar porous	Annealed in air (300 °C, 5 h)	Crystallization (anatase, particle size: 20 nm)	Photocatalysis; Water treatment	2015 [92]
TTIP	He (1 SLM)	He (9 SLM) + O_2_ (0–0.01 SLM)	70–300 W	13.56 MHz	Si (dynamic: 5 mm/s)	-----	He plasma: homogeneous, dense; He + O_2_ plasma: aggregates	-----	-----	Plasma diagnostics	2019 [78]
TIPO ^a^ (0.5 gm/hr)	N_2_ (5 L/min)	N_2_ (30–50 L/min)	600–1000 W	100 kHz	Si (dynamic: 2.5 mm/s)	-----	Crystalline anatase/Agglomerated, hierarchical with cauliflower-like structure	-----	-----	Photocatalysis	2014 [94]
Ti(OtBu)_4_^j^	Ar (4–5.9 mol/hr)	Ar (4–5.9) + O_2_ (0.12–0.8 mol/hr)	200–500 W	13.56 MHz or 2.45 GHz	Glass	-----	Amorphous/uniform, dense, well-adherent	Annealed (440 °C, 2.5 h)	Crystallization (anatase, particle size = 15 nm)	Photocatalysis; Water purification	2002 [34]
TTIP	Ar (1 SLM)	Ar (18 SLM)	370 W	2.45 GHz	Si	-----	Center: crystalline/columnar; Periphery: amorphous/cauliflower-like structure	-----	-----	Plasma diagnostics; Growth mechanism	2017 [90]

^a^ A precursor is introduced into a post-discharge zone. ^b^ A source is moving at the speed of 10–60 mm/s. ^c^ Temperature of substrate (T_sub_) is reported only when external heating is applied and no temperature increase due to plasma interaction is mentioned. ^d^ PD—post-deposition treatment and T_PD_—annealing temperature during PD treatment. ^e^ Only for samples with no substrate heating during plasma interaction. ^f^ Power reported in kV is the applied voltage used to generate plasma. ^g^ Amount of carrier and feed gas introduced is provided in terms of their partial pressures maintained in the deposition chamber. ^h^ n and d_film_ are the refractive index and thickness of the film, respectively. ^i^ TOAA—titanium (IV) oxide acetylacetone (TiO[CH_3_COCH=C(O-)CH_3_]_2_). ^j^ Ti(O*t*Bu)_4_—tiatanium (IV) butoxide. ^k^ A substrate is moving with speed 5 mm/s; therefore, the deposition rate is provided in terms of the thickness of TiO_2_ deposited each time the substrate makes a complete pass through the plasma.

**Table 2:** Summary of deposition and plasma parameters, post-deposition treatment conditions, and their effects on TiO_2_ film morphology for PEALD. Studies are ordered in terms of decreasing plasma pulse time used in each cycle.

Precursor	Carrier Gas (Flow Rate)	Feed Gas (Flow Rate)	Plasma Power ^e^	Frequency	Precursor/Plasma Pulse Time (sec)	Substrate ^c^ (Temperature)	Deposition Rate, nm/Cycle (Conditions)	Film Crystallinity (Conditions, Crystallite Size)/Morphology (Conditions, Particle Size)	PD Treatment (T_PD_, Time) ^d^	Effect of PD Treatment	Scope of Study/Applications	Year [Ref]
TTIP ^a^	N_2_	H_2_O; O_2_; O_3_	160 W	13.56 MHz	15/80: O_2_; 15/30: O_3_	Si (150 °C : O_2_ or O_3_; 275 °C: H_2_O	0.08 (O_2_ plasma)	Crystalline rutile + anatase (dominant)	-----	-----	Instrumentation	2012 [99]
TTIP ^a^	N_2_	O_2_ + Ar (1:1)	100–160 W	13.56 MHz	15/180: O_2_; 15/30 : O_3_	ZnSe; Si (50 °C–150 °C)	0.083	Crystalline rutile + anatase (dominant)	-----	-----	Deposition mechanism	2009 [100]
TDMAT ^h^	N_2_ (60 SCCM)	N_2_ (265 SCCM)	200 W	-----	0.5/30	Si (350 °C)	-----	-----	----	----	Surface modification	2018 [41]
TiCl_4_^a^	Ar (50 SCCM)	H_2_O or O_2_ (5–35) + Ar (50 SCCM)	300 W	13.56 MHz	0.3/3–12	Si (30 °C–180 °C)	0.07–0.08 (300 W, T_sub_ = 90 °C)	Anatase (T_sub_ = 180 °C (H_2_O); ≥90 °C (O_2_))/rough surface	----	----	Synthesis	2016 [98]
TTIP	Ar (150 SCCM)	O_2_ (10–100 SCCM) + H_2_O (as an oxidizer)	100 and 300 W	13.56 MHz	1.5/8 H_2_O pulse: 0.03^g^	Si, fused silica (70 °C–200 °C)	-----	Anatase (low O_2_ flow; 19–30 nm) in amorphous matrix/scattered hillock-like feature with rough and cliffy surface	----	----	Plasma diagnostics	2015 [96]
TTIP	Ar (1000 SCCM)	Ar (1000 SCCM) + O_2_ (0 or 10 SCCM)	2.5–6.5 kV	3–8 kHz	5/7	-----	0.16	-----/Smooth (roughness ~ 0.25 nm)	----	----	Solar cells	2013 [101]
TTIP	-----	O_2_ or N_2_O (30 SCCM)	300 W	-----	1/1 : O_2_ 1/5 : N_2_O	Ru/Ta_2_O_5_/SiO_2_/Si (250 °C)	0.035 (O_2_), 0.059 (N_2_O)	Crystalline rutile/-----spherical particles (30–150 nm)	Annealed in 5% O_2_ + 95% N_2_ (400 °C, 30 mins)	Decrease dielectric constant	DRAM; Electronics	2009 [68]
TiCl_4_^a^	N_2_ (250 SCCM)	O_2_ (50 SCCM)	150 W	13.56 MHz	0.4/3	Si (150 °C)	0.057 (LCM); ^f^ 0.062–0.08 (HCM)	Amorphous/Smooth (LCM); round or conical crystallites in amorphous matrix (HCM)	-----	-----	Plasma diagnostics; Synthesis	2017 [103]
TDEAT ^i^	He	O_2_ (250 SCCM) + Ar (2500 SCCM)	75 W	13.56 MHz	4/2	Si, SiO_2_, TiN (110 °C–350 °C)	0.045 (Si, T_sub_ ≤ 300 °C)	-----	O_2_/Ar/NF_3_ (250/2500/5 SCCM) plasma	Etching (etching rate = 0.19 nm/s)	Synthesis; Electronics	2019 [69]
TTIP; TiCl_4_^a^	N_2_ (250 SCM)	O_2_ (50 SCCM) + N_2_ (250 SCCM)	50–200 W	13.56 MHz	0.1–2/0.1–2	Si (250 °C)	0.092 : TiCl_4_; 0.059 : TTIP (150 W)	Crystalline anatase/granular	-----	-----	Plasma diagnostics	2016 [97]
TDMAT ^h^	Ar (100 SCCM)	O_2_	60 W	-----	0.1/0.5	Si (150 °C–250 °C)	0.036 (200 °C)	-----/Smooth, uniform, continuous (200 °C)	Annealed in O_2_ (500 °C–700 °C, 10 mins)	Increase roughness	Electronics	2004 [67]
TTIP ^a^	N_2_ (25 SCM)	O_2_ (10 SCCM) + N_2_	1–9 kV	60–70 kHz	0.315–1.9/0.0004	Si ^b^	0.15	Amorphous/smooth	-----	-----	Fabrication; Optics	2019 [93]

^a^ A precursor is introduced into a post-discharge zone. ^b^ A source is moving at the speed of 10–60 mm/s. ^c^ Temperature of substrate (T_sub_) is reported only when external heating is applied and no temperature increase due to plasma interaction is mentioned. ^d^ PD—post-deposition treatment and T_PD_—annealing temperature during PD treatment. ^e^ Power reported in kV is the applied voltage used to generate plasma. ^f^ LCM and HCM are low and high current mode, respectively. ^g^ H_2_O pulse was an extra pulse making an ALD cycle made up of precursor/purge/plasma/purge/H_2_O/purge pulses, unlike the common ALD cycle with only precursor/purge/plasma/purge pulses. ^h^ TDMAT—tetrakis(dimethylamido)titanium )IV). ^i^ TDEAT—tetrakis(diehylamido)titanium (IV).

**Table 3:** Summary of deposition and plasma parameters for DBD-based TiO_2_ deposition techniques including deposition rate, crystallinity, and morphology of as-deposited film along with post deposition treatment and its effect on the films.

Precursor	Carrier Gas (Flow Rate)	Feed Gas (Flow Rate)	Plasma Power ^f^	Frequency	Substrate ^c^ (Temperature)	Deposition Rate, nm/min	Film Crystallinity (Conditions, Crystallite Size)/Morphology (Conditions, Particle Size)	PD Treatment (T_PD_, Time) ^d^	Effect of PD Treatment	Scope of Study/Applications	Year [Ref]
**Atmospheric Pressure Dielectric Barrier Discharge**											
TTIP	Ar (2 SLM)	O_2_ (0.5 SLM); Ar (2 SLM) +NH_3_ (NH_3_/Ar = 0.5–5%): N-doping	30 W; 30–100	2.7 kHz	Si (150 °C)	-----	Amorphous/-----	Annealed in air (450 °C, 2 hrs)	Crystallization (anatase)	Photocatalysis; Doping	2019 [43]
TTIP	Ar (0–30 SLM)	O_2_ (0–7.5 SLM) + Ar (0–9.5 SLM)	6–30 W (source)	2.7 kHz	Si (150 °C)	107 (Ar/O_2_: 7.5/0.5 SLM; 15 W)	Amorphous/granular (low flow rate or high power); Dense (high flow rate or low power)	Annealed in air (400 °C, 2 hrs)	Crystallization (anatase + rutile)	Photocatalysis	2018 [107]
-----	-----	Ar (500 SCCM)	40–80 W	13.56 MHz	TiO_2_/quartz	-----	Anatase (power ≥ 60 W)/ columnar (power = 80 W)	----	----	Calcination; Photocatalysis	2019 [76]
TTIP	He (480–500 SCCM)	O_2_ (0–20 SCCM)	14 W	13.56 MHz	Quartz	13 (He + O_2_), 2% of O_2_	Amorphous/He: rough with craters and wrinkles; 2% of O_2_: smooth and transparent; 4% of O_2_: micro-nanoparticles (0.3–3 µm)	----	----	Synthesis	2008 [49]
TiCl_4_	Ar (25 SCCM)	Ar (1000 SCCM) + O_2_ (10 SCCM)	11 KV	10 kHz	Quartz (no heating or 100 °C–400 °C)	1000–3000	Amorphous (no heating); anatase (T_sub_ ≥ 300 °C)/dense and fluffy (T_sub_ ≤ 200 °C); dense cauliflower-like aggregate (T_sub_ ≥ 300 °C)	Annealed in Ar (275 °C, 2 hrs) ^e^	No change in crystallinity	Photocatalysis	2019 [77]
TTIP + H_2_O (10 µL/min)	N_2_ (137.4 SCCM) 0%/100% (1 SLM)	-----	0–10.2 kV	60–240 Hz	Pyrex glass	-----	Anatase (22.2 nm)/nanoparticles (372–27.9 nm at 0–8.58 kV; 60 kHz; TTIP/H_2_O = 11.9); film (240 Hz; 10.2 kV, TTIP/H_2_O = 11.9) spherical particles (30–150 nm)	----	----	Synthesis	2007 [110]
TiO_2_ NPs + HMDSO ^f^	-----	N_2_ (1.2 SLM) + N_2_O (240 SCCM)	-----	0.3–5 kHz	Si	-----	-----	-----	-----	Nanocomposite synthesis	2016 [111]
TTIP	N_2_ (Glass: 25 SCCM) (Si: 53 SCCM)	O_2_ (glass: 0 SCCM) (Si: 0–10 SCCM)	1–9 kV	60–70 kHz	silicate glass; Si ^b^	Glass: 4700–5500 Si: 3250	Amorphous/rough with particle-like features, macroporous (glass); smooth (Si)	-----	-----	Fabrication; Optics	2019 [93]
**Atmospheric Pressure Plasma Jet/Torch**											
-----	-----	Ar (10 L/min) + N_2_ (0.5 L/min)	150 W	10 kHz	TiO_2_/FTO glass	-----	Rutile + anatase (10–50 nm)/porous (25 nm)	-----	-----	DSSCs	2008 [70]
TEOT	He (20 SCCM)	He (300 SCCM) + H_2_ (0–25 SCCM)	105 W	13.56 MHz	Si (200 °C–500 °C)	900 (T_sub_ = 500 °C)	Amorphous (T_sub_ < 350 °C), Crystalline (anatase, >350 °C)	-----	-----	Synthesis; Electronics	1996 [71]
TIOT ^g^	Ar (3000 SCCM)	Ar (4000 SCCM)	18–26 kV	50 kHz	-----	-----	Anatase (dominant) + rutile/cauliflower-like structure with densely aggregated spherical particles with a diameter < 100 nm	-----	-----	Photocatalysis; Hydrophilicity	2020 [109]
TTIP ^a^	Ar (0.2–0.5 L/min)	Ar (5 L/min)	6 kV	50 kHz	Si; Quartz (25 °C–90 °C)	44	Amorphous/rough, granular	-----	-----	Protective coating	2018 [32]
TiCl_4_	O_2_ (25 SCCM)	Ar (6000 SCCM)	30 W	13.56 MHz	FTO glass	9000	Amorphous (25 nm)/porous	Annealed in air (450–500 °C, 1 hr)	Crystallization (anatase)	DSSCs	2010 [64]
**Pulsed Injection Metallorganic Chemical Vapor Deposition**											
TIPO; TOAA ^a^	N_2_ (80%) + O_2_ (20%) (3.5–5 L/min)	N_2_ (80%) + O_2_ (20%) (15 L/min)	600 W	100 kHz	Si (200 °C–300 °C)	6.67	Amorphous (T_sub_ <270 °C), anatase (>270 °C)/smooth, granular (20 nm)	-----	-----	Low temperature synthesis	2008 [72]
**Surface Dielectric Barrier Discharge and Diffuse Coplanar Surface Barrier Discharge**											
TiCl_4_	Ar (14 SCCM)	O_2_ (9 SCCM) + Ar (900 SCCM)	4.2 W	15 kHz	Glass	22	Amorphous/-----	Annealed in air (350–450 °C)	Crystallization (anatase)	Photocatalysis	2009 [65]
-----	-----	Ambient air	400 W	14 kHz	Ti(Bu)/PVP fiber ^h^	-----	-----/Granular	-----	-----	Submicron fiber fabrication	2018 [62]
-----	-----	-----	-----	-----	TiO_2_ + methyl silica binder/FTO	-----	-----	-----	-----	Surface processing; Photocatalysis	2016 [108]

^a^ A precursor is introduced into a post-discharge zone. ^b^ A source is moving at the speed of 10–60 mm/s. ^c^ Temperature of substrate (T_sub_) is reported only when external heating is applied and no temperature increase due to plasma interaction is mentioned. ^d^ PD—post-deposition treatment and T_PD_—annealing temperature during PD treatment. ^e^ Only for samples with no substrate heating during deposition. ^f^ TiO_2_ NPs + HMDSO—TiO_2_ nanoparticles and hexamethyldisiloxane. ^g^ TIOT—tetraisopropylorthotitanate. ^h^ Ti(Bu)/PVP fiber—hybrid titanium butoxide/polyvinylpyrrolidone fiber.

## References

[1] Dowling D.P., Stallard C.P. (2015). Achieving enhanced material finishing using cold plasma treatments. Trans. IMF.

[2] Tendero C., Tixier C., Tristant P., Desmaison J., Leprince P. (2006). Atmospheric pressure plasmas: A review. Spectrochim. Acta Part B Spectrosc..

[3] Chen F.F. (1984). Introduction to Plasma Physics and Controlled Fusion.

[4] Park S., Choe W., Moon S.Y., Yoo S.J. (2019). Electron characterization in weakly ionized collisional plasmas: From principles to techniques. Adv. Phys. X.

[5] Penkov O.V., Khadem M., Lim W.S., Kim D.E. (2015). A review of recent applications of atmospheric pressure plasma jets for materials processing. J. Coat. Technol. Res..

[6] Kakiuchi H., Ohmi H., Yasutake K. (2014). Atmospheric-pressure low-temperature plasma processes for thin film deposition. J. Vac. Sci. Technol. A Vac. Surf. Film..

[7] Schutze A., Jeong J.J.Y., Babayan S.E., Park J., Selwyn G.S., Hicks R.F. (1998). The Atmospheric-Pressure Plasma Jet: A Review and Comparison to Other Plasma Sources. IEEE Trans. Plasma Sci..

[8] Pappas D. (2011). Status and potential of atmospheric plasma processing of materials. J. Vac. Sci. Technol. A Vac. Surf. Film..

[9] Thornton J.A. (1983). Plasma-assisted deposition processes: Theory, mechanisms and applications. Thin Solid Film..

[10] Taylor P.R., Pirzada S.A. (1994). Thermal plasma processing of materials: A review. Adv. Perform. Mater..

[11] Peng X., Matthews A., Xue S. (2011). Plasma-based processes and thin film equipment for nano-scale device fabrication. J. Mater. Sci..

[12] Vallée C., Bonvalot M., Belahcen S., Yeghoyan T., Jaffal M., Vallat R., Chaker A., Lefèvre G., David S., Bsiesy A. (2020). Plasma deposition—Impact of ions in plasma enhanced chemical vapor deposition, plasma enhanced atomic layer deposition, and applications to area selective deposition. J. Vac. Sci. Technol. A.

[13] Fanelli F., Bosso P., Mastrangelo A.M., Fracassi F. (2016). Thin film deposition at atmospheric pressure using dielectric barrier discharges: Advances on three-dimensional porous substrates and functional coatings. Jpn. J. Appl. Phys..

[14] Fauchais P., Vardelle M., Vardelle A., Bianchi L. (1996). Plasma spray: Study of the coating generation. Ceram. Int..

[15] Takahashi Y., Shibata Y., Maeda M., Miyano Y., Murai K., Ohmori A. (2014). Plasma-spraying synthesis of high-performance photocatalytic TiO_2_ coatings. IOP Conf. Ser. Mater. Sci. Eng..

[16] Fakhouri H., Salem D.B., Carton O., Pulpytel J., Arefi-Khonsari F. (2014). Highly efficient photocatalytic TiO_2_ coatings deposited by open air atmospheric pressure plasma jet with aerosolized TTIP precursor. J. Phys. D Appl. Phys..

[17] Chen D., Jordan E.H., Gell M. (2008). Porous TiO_2_ coating using the solution precursor plasma spray process. Surf. Coat. Technol..

[18] Chen D., Jordan E.H., Gell M., Ma X. (2008). Dense TiO_2_ Coating Using the Solution Precursor Plasma Spray Process. J. Am. Ceram. Soc..

[19] Bessergenev V.G., Khmelinskii I.V., Pereira R.J.F., Krisuk V.V., Turgambaeva A.E., Igumenov I.K. (2002). Preparation of TiO_2_ films by CVD method and its electrical, structural and optical properties. Vacuum.

[20] O’Neill S.A., Parkin I.P., Clark R.J.H., Mills A., Elliott N. (2003). Atmospheric pressure chemical vapour deposition of titanium dioxide coatings on glass. J. Mater. Chem..

[21] Kanazawa T., Ohmori A. (2005). Behavior of TiO_2_ coating formation on PET plate by plasma spraying and evaluation of coating’s photocatalytic activity. Surf. Coat. Technol..

[22] Jung H., Park J., Yoo E.S., Han G.-S., Jung H.S., Ko M.J., Park S., Choe W. (2013). Functionalization of nanomaterials by non-thermal large area atmospheric pressure plasmas: Application to flexible dye-sensitized solar cells. Nanoscale.

[23] Guo X., Ma T.P. (1998). Tunneling leakage current in oxynitride: Dependence on oxygen/nitrogen content. IEEE Electron Device Lett..

[24] Fujishima A., Rao T.N., Tryk D.A. (2000). Titanium dioxide photocatalysis. J. Photochem. Photobiol. C Photochem. Rev..

[25] Malagutti A.R., Mourão H.A.J.L., Garbin J.R., Ribeiro C. (2009). Deposition of TiO_2_ and Ag:TiO_2_ thin films by the polymeric precursor method and their application in the photodegradation of textile dyes. Appl. Catal. B Environ..

[26] Luttrell T., Halpegamage S., Tao J., Kramer A., Sutter E., Batzill M. (2015). Why is anatase a better photocatalyst than rutile?—Model studies on epitaxial TiO_2_ films. Sci. Rep..

[27] Davidsdóttir S., Canulescu S., Dirscherl K., Schou J., Ambat R. (2013). Investigation of photocatalytic activity of titanium dioxide deposited on metallic substrates by DC magnetron sputtering. Surf. Coat. Technol..

[28] Carp O., Huisman C.L., Reller A. (2004). Photoinduced reactivity of titanium dioxide. Prog. Solid State Chem..

[29] Augugliaro V., Loddo V., Pagliaro M., Palmisano G., Palmisano L. (2011). Clean by Light Irradiation: Practical Applications of Supported TiO_2_.

[30] Fujishima A., Zhang X., Tryk D.A. (2008). TiO_2_ photocatalysis and related surface phenomena. Surf. Sci. Rep..

[31] Fujishima A., Hashimoto K., Watanabe T. (1999). TiO_2_ Photocatalysis: Fundamentals and Applications.

[32] Xu J., Nagasawa H., Kanezashi M., Tsuru T. (2018). UV-Protective TiO_2_ Thin Films with High Transparency in Visible Light Region Fabricated via Atmospheric-Pressure Plasma-Enhanced Chemical Vapor Deposition. ACS Appl. Mater. Interfaces.

[33] Evans P., Sheel D.W. (2007). Photoactive and antibacterial TiO_2_ thin films on stainless steel. Surf. Coat. Technol..

[34] Karches M., Morstein M., von Rohr R.P., Pozzo R.L., Giombi J.L., Baltanás M.A. (2002). Plasma-CVD-coated glass beads as photocatalyst for water decontamination. Catal. Today.

[35] Fagan R., McCormack D.E., Dionysiou D.D., Pillai S.C. (2016). A review of solar and visible light active TiO_2_ photocatalysis for treating bacteria, cyanotoxins and contaminants of emerging concern. Mater. Sci. Semicond. Process..

[36] Thukkaram M., Cools P., Nikiforov A., Rigole P., Coenye T., Van Der Voort P., Du Laing G., Vercruysse C., Declercq H., Morent R. (2020). Antibacterial activity of a porous silver doped TiO_2_ coating on titanium substrates synthesized by plasma electrolytic oxidation. Appl. Surf. Sci..

[37] Lee D.G., Lee D., Yoo J.S., Lee S., Jung H.S. (2016). Effective passivation of Ag nanowire-based flexible transparent conducting electrode by TiO_2_ nanoshell. Nano Converg..

[38] Schneider J., Matsuoka M., Takeuchi M., Zhang J., Horiuchi Y., Anpo M., Bahnemann D.W. (2014). Understanding TiO_2_ Photocatalysis: Mechanisms and Materials. Chem. Rev..

[39] Asahi R., Morikawa T., Ohwaki T., Aoki K., Taga Y. (2001). Visible-Light Photocatalysis in Nitrogen-Doped Titanium Oxides. Science.

[40] Hovish M.Q., Dauskardt R.H. (2016). Optical properties of metal oxynitride thin films grown with atmospheric plasma deposition. J. Phys. D Appl. Phys..

[41] Kot M., Łobaza J., Naumann F., Gargouri H., Henkel K., Schmeißer D. (2018). Long-term ambient surface oxidation of titanium oxynitride films prepared by plasma-enhanced atomic layer deposition: An XPS study. J. Vac. Sci. Technol. A.

[42] Hovish M.Q., Hilt F., Rolston N., Xiao Q., Dauskardt R.H. (2019). Open Air Plasma Deposition of Superhydrophilic Titania Coatings. Adv. Fun. Mat..

[43] Chen Q., Ozkan A., Chattopadhyay B., Baert K., Poleunis C., Tromont A., Snyders R., Delcorte A., Terryn H., Delplancke-Ogletree M.-P. (2019). N-Doped TiO_2_ Photocatalyst Coatings Synthesized by a Cold Atmospheric Plasma. Langmuir.

[44] Haidera A.J., Jameel Z.N., Al-Hussainib I.H.M. (2019). Review on: Titanium Dioxide Applications. Energy Procedia.

[45] Kumaravel V., Mathew S., Bartlett J., Pillaiab S.C. (2019). Photocatalytic hydrogen production using metal doped TiO_2_: A review of recent advances. App. Catal. B.

[46] Rajaraman T.S., Parikh S.P., Gandhi V.G. (2020). Black TiO_2_: A review of its properties and conflicting trends. Chem. Eng. J..

[47] Noman M.T., Ashraf M.A., Ali A. (2019). Synthesis and applications of nano-TiO_2_: A review. Environ. Sci. Pollut. Res..

[48] Martinu L., Poitras D. (2000). Plasma deposition of optical films and coatings: A review. J. Vac. Sci. Technol. A Vac. Surf. Film..

[49] Yoshiki H., Saito T. (2008). Preparation of TiO_2_ thin films on the inner surface of a quartz tube using atmospheric-pressure microplasma. J. Vac. Sci. Technol. A Vac. Surf. Film..

[50] Filippin A.N., Sanchez-Valencia J.R., Idígoras J., Rojas T.C., Barranco A., Anta J.A., Borras A. (2017). Plasma enhanced deposition of single and multistacked TiO_2_ hierarchical nanotube photoanodes. Nanoscale.

[51] Gazal Y., Dublanche-Tixier C., Chazelas C., Colas M., Carles P., Tristant P. (2016). Multi-structural TiO_2_ film synthesised by an atmospheric pressure plasma-enhanced chemical vapour deposition microwave torch. Thin Solid Film..

[52] Yamauchi S., Imai Y. (2013). Plasma-Assisted Chemical Vapor Deposition of TiO_2_ Thin Films for Highly Hydrophilic Performance. Cryst. Struct. Theory Appl..

[53] Wang S., Wang K., Jehng J., Liu L. (2012). Preparation of TiO_2_/MCM-41 by plasma enhanced chemical vapor deposition method and its photocatalytic activity. Front. Environ. Sci. Eng..

[54] Yang W., Wolden C.A. (2006). Plasma-enhanced chemical vapor deposition of TiO_2_ thin films for dielectric applications. Thin Solid Film..

[55] Dalapati G.K., Chatterjee S., Samanta S.K., Maiti C.K. (2003). Electrical characterization of low temperature deposited TiO_2_ films on strained-SiGe layers. Appl. Surf. Sci..

[56] Lee G.W., Woo S.I., Kim J.C., Oh K.H. (1994). Preparation and properties of amorphous TiO_2_ thin films by plasma enhanced chemical vapor deposition. Thin Solid Film..

[57] Frenck H.J., Kulisch W., Kuhr M., Kassing R. (1991). Deposition of TiO_2_ thin films by plasma-enhanced decompostion of tetraisopropyltitanate. Thin Solid Film..

[58] Borras A., Sanchez-Valencia J.R., Widmer R., Rico V.J., Justo A., Gonzalez-Elipe A.R. (2009). Growth of Crystalline TiO_2_ by Plasma Enhanced Chemical Vapor Deposition. Cryst. Growth Des..

[59] Lee Y.H. (1998). A role of energetic ions in RF-biased PECVD of TiO_2_. Vacuum.

[60] Maiti C.K., Samanta S.K., Dalapati G.K., Nandi S.K., Chatterjee S. (2004). Electrical characterization of TiO_2_ gate oxides on strained-Si. Microelectron. Eng..

[61] Hao Q., Fu X., Song S., Gibson D., Li C., Chu H.O., Shi Y. (2018). Investigation of TiO_2_ Thin Film Deposited by Microwave Plasma Assisted Sputtering and Its Application in 3D Glasses. Coatings.

[62] Medvecká V., Kováčik D., Zahoranová A., Černák M. (2018). Atmospheric pressure plasma enhanced calcination by the preparation of TiO_2_ fibers in submicron scale. Appl. Surf. Sci..

[63] Mertens J., Hubert J., Vandencasteele N., Raes M., Terryn H., Reniers F. (2017). Chemical and physical effect of SiO_2_ and TiO_2_ nanoparticles on highly hydrophobic fluorocarbon hybrid coatings synthesized by atmospheric plasma. Surf. Coat. Technol..

[64] Seo H.K., Elliott C.M., Shin H.S. (2010). Mesoporous TiO_2_ films fabricated using atmospheric pressure dielectric barrier discharge jet. ACS Appl. Mater. Interfaces.

[65] Di L.B., Li X.S., Shi C., Xu Y., Zhao D.Z., Zhu A.M. (2009). Atmospheric-pressure plasma CVD of TiO_2_ photocatalytic films using surface dielectric barrier discharge. J. Phys. D Appl. Phys..

[66] Fanelli F., Fracassi F. (2017). Atmospheric pressure non-equilibrium plasma jet technology: General features, specificities and applications in surface processing of materials. Surf. Coat. Technol..

[67] Park J.J., Lee W.J., Lee G.H., Kim I.S., Shin B.C., Yoon S.G. (2004). Very thin TiO_2_ films Prepared by Plasma Enhanced Atomic Layer Deposition (PEALD). Integr. Ferroelectr..

[68] Choi G., Kim S.K., Won S., Kim H.J., Hwang C.S. (2009). Plasma-Enhanced Atomic Layer Deposition of TiO_2_ and Al-Doped TiO_2_ Films Using N_2_O and O_2_ Reactants. J. Electrochem. Soc..

[69] Vallat R., Gassilloud R., Salicio O., El Hajjam K., Molas G., Pelissier B., Vallée C. (2019). Area selective deposition of TiO_2_ by intercalation of plasma etching cycles in PEALD process: A bottom up approach for the simplification of 3D integration scheme. J. Vac. Sci. Technol. A.

[70] Yuji T., Akatsuka H., Mungkung N., Park B.W., Sung Y.M. (2009). Surface treatment of TiO_2_ films for dye-sensitized solar cells using atmospheric-pressure non-equilibrium DC pulse discharge plasma jet. Vacuum.

[71] Ha H., Yoshimoto M., Koinuma H., Moon B., Ishiwara H. (1996). Open air plasma chemical vapor deposition of highly dielectric amorphous TiO_2_ films. Appl. Phys. Lett..

[72] Jiménez C., De Barros D., Darraz A., Deschanvres J.-L., Rapenne L., Chaudouët P., Méndez J.E., Weiss F., Thomachot M., Sindzingre T. (2007). Deposition of TiO_2_ thin films by atmospheric plasma post-discharge assisted injection MOCVD. Surf. Coat. Technol..

[73] Williams L.M., Hess D.W. (1983). Structural properties of titanium dioxide films deposited in an rf glow discharge. J. Vac. Sci. Technol. A.

[74] Williams L.M., Hess D.W. (1984). Photoelectrochemical properties of plasma-deposited TiO_2_ thin films. Thin Solid Film..

[75] Collette S., Hubert J., Batan A., Baert K., Raes M., Vandendael I., Daniel A., Archambeau C., Terryn H., Reniers F. (2016). Photocatalytic TiO_2_ thin films synthesized by the post-discharge of an RF atmospheric plasma torch. Surf. Coat. Technol..

[76] Xu Y., Zhang Y., He T., Ding K., Huang X., Li H., Shi J., Guo Y., Zhang J. (2019). The Effects of Thermal and Atmospheric Pressure Radio Frequency Plasma Annealing in the Crystallization of TiO_2_ Thin Films. Coatings.

[77] Xu Y., Zhang Y., Li L., Ding K., Guo Y., Shi J., Huang X., Zhang J. (2019). Synergistic Effect of Plasma Discharge and Substrate Temperature in Improving the Crystallization of TiO_2_ Film by Atmospheric Pressure Plasma Enhanced Chemical Vapor Deposition. Plasma Chem. Plasma Process..

[78] Mauchauffé R., Kang S., Kim J., Kim J.H., Moon S.Y. (2019). Spectroscopic study of an atmospheric pressure plasma generated for the deposition of titanium dioxide thin films. Curr. Appl. Phys..

[79] Huang S.S., Chen J.S. (2002). Comparison of the characteristics of TiO_2_ films prepared by low-pressure and plasma-enhanced chemical vapor deposition. J. Mater. Sci. Mater. Electron..

[80] Nakamura M., Kato S., Aoki T., Sirghi L., Hatanaka Y. (2001). Formation mechanism for TiO_x_ thin film obtained by remote plasma enhanced chemical vapor deposition in H_2_–O_2_ mixture gas plasma. Thin Solid Film..

[81] Mathur S., Kuhn P. (2006). CVD of titanium oxide coatings: Comparative evaluation of thermal and plasma enhanced processes. Surf. Coat. Technol..

[82] Rie K.T., Gebauer A., Wohle J. (1996). Plasma assisted CVD for low temperature coatings to improve the wear and corrosion resistance. Surf. Coat. Technol..

[83] Kang S., Mauchauffé R., You Y.S., Moon S.Y. (2018). Insights into the Role of Plasma in Atmospheric Pressure Chemical Vapor Deposition of Titanium Dioxide Thin Films. Sci. Rep..

[84] Baba K., Bulou S., Choquet P., Boscher N.D. (2017). Photocatalytic Anatase TiO_2_ Thin Films on Polymer Optical Fiber Using Atmospheric-Pressure Plasma. ACS Appl. Mater. Interfaces.

[85] Ahn K.H., Park Y.B., Park D.W. (2003). Kinetic and mechanistic study on the chemical vapor deposition of titanium dioxide thin films by in situ FT-IR using TTIP. Surf. Coat. Technol..

[86] Rong W., Kazuhito H., Akira F., Makoto C., Eiichi K., Astushi K., Mitsuhide S., Toshiya W. (1997). Light-induced amphiphilic surfaces. Nature.

[87] Battistona G.A., Gerbasia R., Gregoria A., Porchiaa M., Cattarinb S., Rizzic G.A. (2000). PECVD of amorphous TiO_2_ thin films: Effect of growth temperature and plasma gas composition. Thin Solid Film..

[88] Zhou M., Roualdès S., Zhao J., Autès V., Ayral A. (2015). Nanocrystalline TiO_2_ thin film prepared by low-temperature plasma-enhanced chemical vapor deposition for photocatalytic applications. Thin Solid Film..

[89] Perraudeau A., Dublanche-Tixier C., Tristant P., Chazelas C., Vedraine S., Ratier B. (2019). Low-temperature deposition of TiO_2_ by atmospheric pressure PECVD towards photoanode elaboration for perovskite and solid-state dye-sensitized solar cells. EPJ Photovolt..

[90] Gazal Y., Chazelas C., Dublanche-Tixier C., Tristant P. (2017). Contribution of optical emission spectroscopy measurements to the understanding of TiO_2_ growth by chemical vapor deposition using an atmospheric-pressure plasma torch. J. Appl. Phys..

[91] Perraudeau A., Dublanche-Tixier C., Tristant P., Chazelas C. (2019). Dynamic mode optimization for the deposition of homogeneous TiO_2_ thin film by atmospheric pressure PECVD using a microwave plasma torch. Appl. Surf. Sci..

[92] Zhou M., Roualdès S., Ayral A. (2015). New photocatalytic contactors obtained by PECVD deposition of TiO_2_ thin layers on the surface of macroporous supports: PECVD TiO_2_-based membranes as photocatalytic contactors. Eur. Phys. J. Spec. Top..

[93] Aghaee M., Verheyen J., Stevens A.A.E., Kessels W.M.M., Creatore M. (2019). TiO_2_ thin film patterns prepared by chemical vapor deposition and atomic layer deposition using an atmospheric pressure microplasma printer. Plasma Process. Polym..

[94] Boscher N.D., Olivier S., Maurau R., Bulou S., Sindzingre T., Belmonte T., Choquet P. (2014). Photocatalytic anatase titanium dioxide thin films deposition by an atmospheric pressure blown arc discharge. Appl. Surf. Sci..

[95] Profijt H.B., van de Sanden M.C.M., Kessels W.M.M. (2012). Substrate-biasing during plasma-assisted atomic layer deposition to tailor metal-oxide thin film growth. J. Vac. Sci. Technol. A Vac. Surf. Film..

[96] Ratzsch S., Kley E.B., Tünnermann A., Szeghalmi A. (2015). Influence of the oxygen plasma parameters on the atomic layer deposition of titanium dioxide. Nanotechnology.

[97] Chiappim W., Testoni G.E., Doria A.C.O.C., Pessoa R.S., Fraga M.A., Galvão N.K.A.M., Grigorov K.G., Vieira L., Maciel H.S. (2016). Relationships among growth mechanism, structure and morphology of PEALD TiO_2_ films: The influence of O_2_ plasma power, precursor chemistry and plasma exposure mode. Nanotechnology.

[98] Strobel A., Schnabel H.-D., Reinhold U., Rauer S., Neidhardt A. (2016). Room temperature plasma enhanced atomic layer deposition for TiO_2_ and WO_3_ films. J. Vac. Sci. Technol. A.

[99] Rai V.R., Agarwal S. (2012). In situ diagnostics for studying gas-surface reactions during thermal and plasma-assisted atomic layer deposition. J. Vac. Sci. Technol. A.

[100] Rai V.R., Agarwal S. (2009). Surface reaction mechanisms during plasma-assisted atomic layer deposition of titanium dioxide. J. Phys. Chem. C.

[101] Theirich D., Müller R., Zilberberg K., Trost S., Behrendt A., Riedl T. (2013). Atmospheric pressure plasma ALD of titanium oxide. Chem. Vap. Depos..

[102] Lisovskiy V.A., Yegorenkov V.D. (2004). Alpha–gamma transition in RF capacitive discharge in low-pressure oxygen. Vacuum.

[103] Napari M., Tarvainen O., Kinnunen S., Arstila K., Julin J., Fjellvåg Ø.S., Weibye K., Nilsen O., Sajavaara T. (2017). The α and γ plasma modes in plasma-enhanced atomic layer deposition with O_2_–N_2_ capacitive discharges. J. Phys. D.

[104] Profijt H.B., Potts S.E., van de Sanden M.C.M., Kessels W.M.M. (2011). Plasma-Assisted Atomic Layer Deposition: Basics, Opportunities, and Challenges. J. Vac. Sci. Technol. A.

[105] Knoops H.C.M., Faraz T., Arts K., Kessels W.M.M. (2019). Status and prospects of plasma-assisted atomic layer deposition featured. J. Vac. Sci. Technol. A.

[106] Niemelä J.P., Marin G., Karppinen M. (2017). Titanium dioxide thin films by atomic layer deposition: A review. Semicond. Sci. Technol..

[107] Chen Q., Liu Q., Ozkan A., Chattopadhyay B., Wallaert G., Baert K., Terryn H., Delplancke-Ogletree M.P., Geerts Y., Reniers F. (2018). Atmospheric pressure dielectric barrier discharge synthesis of morphology-controllable TiO_2_ films with enhanced photocatalytic activity. Thin Solid Film..

[108] Homola T., Dzik P., Veselý M., Kelar J., Černák M., Weiter M. (2016). Fast and Low-Temperature (70 °C) Mineralization of Inkjet Printed Mesoporous TiO_2_ Photoanodes Using Ambient Air Plasma. ACS Appl. Mater. Interfaces.

[109] Pandiyaraj K.N., Vasu D., Padmanabhan P.V.A., Ghobeira R., Tabaei P.S.E., Coolsb P., De Geyter N., Morent R., Deshmukh R.R., Pichumanid M. (2020). Synergetic effect of the catalytic action of plasma jet deposited TiO_x_ coatings and atmospheric pressure plasma treatment on the degradation of RYRR. Surf. Coat. Technol..

[110] Chen C., Bai H., Chein H.M., Chen T.M. (2007). Continuous generation of TiO_2_ nanoparticles by an atmospheric pressure plasma-enhanced process. Aerosol Sci. Technol..

[111] Profili J., Gherardi N., Naudé N., Stafford L. (2016). Deposition of TiO_2_-SiO_2_ nanocomposite coatings using atmospheric-pressure plasmas. Proceedings of the IEEE Nanotechnology Materials and Devices Conference.

[112] Peng T., Pulpytel J., Horovitz I., Jaiswal A.K., Avisar D., Mamane H., Lalman J.A., Arefi-Khonsari F. (2019). One-step deposition of nano-Ag-TiO_2_ coatings by atmospheric pressure plasma jet for water treatment: Application to trace pharmaceutical removal using solar photocatalysis. Plasma Process. Polym..

[113] Liao W.Y., Yang Y.J., Hsu C.M., Hsu C.C., Cheng I.C., Chen J.Z. (2015). Atmospheric-pressure-plasma-jet sintered dual-scale porous TiO_2_ using an economically favorable NaCl solution. J. Power Sources.

[114] Chang H., Yang Y.-J., Hsu C.-M., Hsu C.-C., Cheng I.-C., Chen J.-Z. (2014). Atmospheric-Pressure-Plasma-Jet Particulate TiO_2_ Scattering Layer Deposition Processes for Dye-Sensitized Solar Cells. ECS J. Solid State Sci. Technol..

[115] Chou C.-Y., Chang H., Liu H.-W., Yang Y.-J., Hsu C.-C., Cheng I.-C., Chen J.-Z. (2015). Atmospheric-pressure-plasma-jet processed nanoporous TiO_2_ photoanodes and Pt counter-electrodes for dye-sensitized solar cells. RSC Adv..

[116] Chen J.-Z., Hsu C.-C., Wang C., Liao W.-Y., Wu C.-H., Wu T.-J., Liu H.-W., Chang H., Lien S.-T., Li H.-C. (2015). Rapid Atmospheric-Pressure-Plasma-Jet Processed Porous Materials for Energy Harvesting and Storage Devices. Coatings.

[117] Liu Z., Chen Q., Wang Z., Yang L., Wang C. (2011). Production of titanium dioxide powders by atmospheric pressure plasma jet. Phys. Procedia.

[118] Krüger F., Gergs T., Trieschmann J. (2019). Machine learning plasma-surface interface for coupling sputtering and gas phase transport simulations. Plasma Sources Sci. Technol..

[119] Gidon D., Pei X., Bonzanini A.D., Graves D.B., Mesbah A. (2019). Machine Learning for Real-Time Diagnostics of Cold Atmospheric Plasma Sources. IEEE Trans. Radiat. Plasma Med. Sci..

[120] Pakseresht A.H., Ghasali E., Nejati M., Shirvanimoghaddam K., Javadi A.H., Teimouri R. (2015). Development empirical-intelligent relationship between plasma spray parameters and coating performance of yttria-stabilized zirconia. Int. J. Adv. Manuf. Technol..

